# Interplay between transforming growth factor-β and Nur77 in dual regulations of inhibitor of differentiation 1 for colonic tumorigenesis

**DOI:** 10.1038/s41467-021-23048-5

**Published:** 2021-05-14

**Authors:** Boning Niu, Jie Liu, Ben Lv, Jiacheng Lin, Xin Li, Chunxiao Wu, Xiaohua Jiang, Zhiping Zeng, Xiao-kun Zhang, Hu Zhou

**Affiliations:** 1 School of Pharmaceutical Sciences, Fujian Provincial Key Laboratory of Innovative Drug Target Research, High Throughput Drug Screening Platform, Xiamen University, Xiamen, Fujian China; 2 School of Biomedical Sciences, The Chinese University of Hong Kong, Hong Kong, China

**Keywords:** Cancer, Cell biology, Oncology

## Abstract

The paradoxical roles of transforming growth factor-β (TGFβ) signaling and nuclear receptor Nur77 in colon cancer development are known but the underlying mechanisms remain obscure. Inhibitor of differentiation 1 (ID1) is a target gene of TGFβ and a key promoter for colon cancer progression. Here, we show that Nur77 enhances TGFβ/Smad3-induced ID1 mRNA expression through hindering Smurf2-mediated Smad3 mono-ubiquitylation, resulting in ID1 upregulation. In the absence of TGFβ, however, Nur77 destabilizes ID1 protein by promoting Smurf2-mediated ID1 poly-ubiquitylation, resulting in ID1 downregulation. Interestingly, TGFβ stabilizes ID1 protein by switching Nur77 interaction partners to inhibit ID1 ubiquitylation. This also endows TGFβ with an active pro-tumorigenic action in Smad4-deficient colon cancers. Thus, TGFβ converts Nur77’s role from destabilizing ID1 protein and cancer inhibition to inducing ID1 mRNA expression and cancer promotion, which is highly relevant to colon cancer stemness, metastasis and oxaliplatin resistance. Our data therefore define the integrated duality of Nur77 and TGFβ signaling in regulating ID1 expression and provide mechanistic insights into the paradoxical roles of TGFβ and Nur77 in colon cancer progression.

## Abstract (web summary)

Inhibitor of Differentiation 1 (ID1) is an oncogene for colorectal cancer. Here, the authors show a complex interplay between nuclear receptor Nur77 and Transforming Growth Factor-β (TGFβ) to regulate ID1 expression at both transcriptional and post-translational levels which is relevant to colon cancer stemness, metastasis and resistance to oxaliplatin.

## Introduction

The process of colon cancer development is sequentially controlled by different signaling pathways, of which transforming growth factor-β (TGFβ) signaling plays a unique role by imposing opposite influence at different stages of cancer developments^1–4^. In early-stage cancer, TGFβ exerts tumor suppressor functions including cell-cycle arrest and apoptotic induction. However, it promotes tumorigenesis including metastasis and chemoresistance in late-stage cancer^5^. In the canonical TGFβ signal transduction, TGFβ binds to TGFβ type II receptor (TβRII) on the plasma membrane and then recruits and activates type I receptor (TβRI)^6^. Activated TβRI phosphorylates cytoplasmic Smad2 and Smad3, leading to their association with Smad4 to form a complex that translocates into the nucleus and binds the cognate DNA elements to regulate gene transcription^7–9^.

Although TGFβ promotes cancer progression at late stages, a substantial fraction of high-grade colon cancers lacks canonical TGFβ signaling, largely due to mutations in receptors or Smad4^10–13^. The pro-tumorigenic effect of TGFβ in Smad4-deficient cancers remains incompletely understood, but the loss of Smad4 may allow cancer cells to escape TGFβ-mediated growth arrest and immune surveillance^9,14–18^. The above loss-of-function mechanisms reflect a passive action mode of TGFβ signaling in cancer promotion. Whether TGFβ actively promotes cancer progression in Smad4-deficient colon cancers is not well established.

Aberrantly high expression of inhibitor of differentiation 1 (ID1) contributes to the growth, self-renewal, and metastasis of a variety of tumors including colon cancer^19–22^. ID1 acts mainly through binding the helix–loop–helix transcription factors to prevent their induction of differentiation genes^23,24^. In cancer cells, ID1 transcription is upregulated by TGFβ/Smad3 signaling^25^. Smad-specific E3 ubiquitin-protein ligase 2 (Smurf2) catalyzes Smad3 multiple mono-ubiquitylation to inhibit its ability to activate transcription^26^. Interestingly, Smurf2 also mediates ID1 poly-ubiquitylation and degradation^27^. However, it remains unclear how these diverse functions of Smurf2 are regulated.

Nur77, an orphan member of the nuclear receptor superfamily, plays a critical role in numerous biological processes such as growth, survival, differentiation, and apoptosis in response to diverse intracellular and extracellular stimuli^28,29^. Besides transcriptional regulation, Nur77 possesses non-genomic activities such as localizing on the mitochondria to regulate apoptosis and mitophagy^28,30,31^. Nur77 was reported to exert both tumor-suppressing and tumor-promoting effects in colon cancers^32^. It has been identified as a positive regulator of TGFβ signaling involved in breast cancer progression^33^. Whether Nur77 regulates the TGFβ/ID1 axis and why Nur77 plays an opposite role in colon cancer development remain elusive.

Here, we reveal an unanticipated crosstalk between Nur77 and TGFβ signaling in regulating ID1 expression at both transcriptional and post-translational levels, which is pathophysiologically relevant to colon cancer development. Our data provide mechanistic insight into the opposite role of Nur77 in colon cancers and unravels the active mode of TGFβ for tumorigenesis in Smad4-deficient colon cancers.

## Results

### Nur77 transcriptionally upregulates TGFβ-induced ID1 expression through inhibiting Smurf2-mediated mono-ubiquitylation of Smad3

**Fig. 1:**
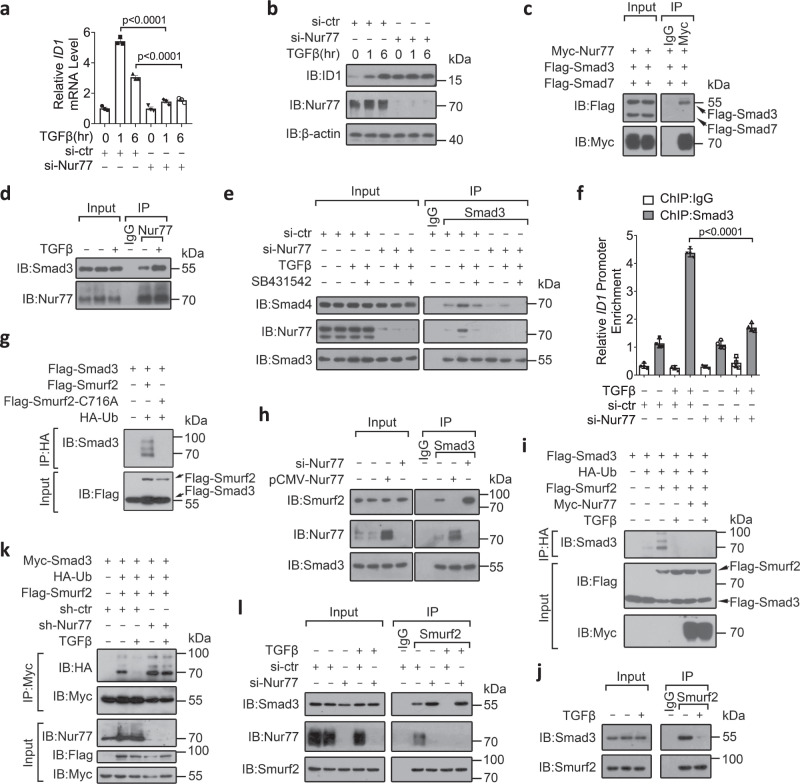
Nur77 transcriptionally upregulates TGFβ-induced ID1 expression through inhibiting Smurf2-mediated mono-ubiquitylation of Smad3. **a**, **b** LS174T cells transfected with the indicated siRNAs for 48 h were treated with TGFβ (10 ng/mL) for the indicated time. ID1 mRNA and protein expressions were examined by qRT-PCR (**a**) and immunoblotting (IB) (**b**), respectively. si-ctr control siRNA, si-Nur77 Nur77 siRNA, hr hour. Two-way ANOVA followed by Tukey’s multiple comparisons test was used for statistical analysis, and data are presented as means ± SD (*n* = 3 biologically independent samples). **c**–**e** HCT116 cells were transfected with the indicated expression plasmids for 24 h or siRNAs for 48 h before SB431542 (10 μM) treatment for 1 h. Cells were then treated with TGFβ (10 ng/mL) for 1 h. Protein interactions were examined by co-immunoprecipitation (co-IP) using specific antibodies. IgG control IgG. **f**–**l** LS174T cells were transfected with the indicated expression plasmids, siRNAs, or shRNAs before TGFβ (10 ng/mL) treatment for 1 h. Chromatin immunoprecipitations were performed using control IgG or anti-Smad3 antibody followed by PCR (**f**). Smad3 ubiquitylation was examined by IP and IB with specific antibodies (**g**, **i**, **k**). Protein interactions were examined by co-IP (**h**, **j**, **l**). sh-ctr control shRNA, sh-Nur77 Nur77 shRNA, Ub ubiquitin. Two-way ANOVA followed by Tukey’s multiple comparisons test was used for statistical analysis, and data are presented as means ± SD (*n* = 4 biologically independent samples). Data represent at least two independent experiments. Source data are provided as Source Data file.

To explore the potential regulation of TGFβ signaling by nuclear receptors, we examined the effect of ectopically expressed nuclear receptors on regulating TGFβ/Smad3-responsive CAGA reporter activity^34^. Among six nuclear receptors examined, only Nur77 significantly enhanced TGFβ-induced CAGA reporter activity in both HEK293T kidney and HCT116 colon cancer cells (Supplementary Fig. 1a, b), indicating the positive role of Nur77 in TGFβ/Smad3 signaling. We next investigated the effect of Nur77 on TGFβ-induced expression of ID1, a key TGFβ/Smad3 target gene known to mediate the oncogenic activity of TGFβ^35^. TGFβ substantially induced ID1 messenger RNA (mRNA) and protein expression in LS174T colon cancer cells. This effect of TGFβ depended on Nur77 and Smad3 as it was potently suppressed by small interference RNA (siRNA)-mediated knockdown of these two factors (Fig. 1a, b and Supplementary Fig. 1c). Thus, Nur77 potentiates the effect of TGFβ on ID1 mRNA induction.

Nur77 has been reported to bind Smad7, an inhibitory Smad in TGFβ signaling, and induce its degradation, resulting in the enhancement of TGFβ signaling in breast cancer^33^. However, we failed to observe significant alterations in Smad7 expression and Smad2/3 phosphorylation upon overexpression or suppression of Nur77 in colon cancer cells (Supplementary Fig. 1d), suggesting a distinct mode of Nur77 action in colon cancers. We then examined the interaction of Nur77 with other Smads involved in TGFβ signaling. Intriguingly, Nur77 potently interacted with Smad3 while it showed weak binding to Smad2 and no binding to Smad4 (Supplementary Fig. 1e). In HCT116 cells, Nur77 interacted with Smad3 but not Smad7 under identical conditions (Fig. 1c). Both the endogenous and exogenous interactions of Nur77 and Smad3 were enhanced by TGFβ (Fig. 1d and Supplementary Fig. 1f). Nur77 suppression mimicked the effect of TβRI antagonist SB431542^36^, inhibiting TGFβ-induced Smad3/Smad4 complex formation (Fig. 1e). Furthermore, Nur77 enhanced TGFβ-induced Smad3 binding to the *ID1* gene promoter (Fig. 1f and Supplementary Fig. 1g). Collectively, these results suggest that Nur77 binds Smad3 to enhance its association with Smad4 and the *ID1* promoter, promoting TGFβ-induced *ID1* gene transcription.

The multiple mono-ubiquitylation of Smad3 mediated by Smurf2, an E3 ubiquitin ligase, has been shown to block Smad3/Smad4 complex formation^26^. In agreement, Smurf2 but not Smurf2^C716A^, a catalytically inactive mutant of Smurf2^37^, induced Smad3 ubiquitylation in LS174T cells, although mutant Smurf2^C716A^ bound to Smad3 with the same efficiency as Smurf2 (Fig. 1g and Supplementary Fig. 1h). Smurf2-induced Smad3 ubiquitylation was not affected by two ubiquitin mutants (K48R and K63R) in which lysines 48 or 63 were mutated to arginine (Supplementary Fig. 1i), suggesting that Smurf2 catalyzed multiple mono-ubiquitylation of Smad3. Interestingly, the basal and Smurf2-mediated Smad3 ubiquitylation was largely blocked by Nur77 overexpression (Supplementary Fig. 1i, j). Mechanistically, Nur77 inhibited the interaction of Smurf2 with Smad3 (Fig. 1h and Supplementary Fig. 1h). Interestingly, TGFβ inhibited not only the basal Smad3 ubiquitylation in a dose-dependent manner but also Smurf2-mediated Smad3 ubiquitylation (Supplementary Fig. 1k and Fig. 1i), likely through inhibiting the association of Smurf2 with Smad3 (Fig. 1j and Supplementary Fig. 1l). Moreover, Nur77 was found to mediate the inhibitory effects of TGFβ on Smad3 ubiquitylation and Smurf2–Smad3 association (Fig. 1k, l). Thus, it appears that Nur77 enhances TGFβ-induced Smad3/Smad4 complex formation through binding Smad3 to inhibit Smurf2 interacting with and mono-ubiquitylating Smad3.

### Nur77 post-translationally downregulates ID1 through mediating its association with and poly-ubiquitylation by Smurf2

**Fig. 2:**
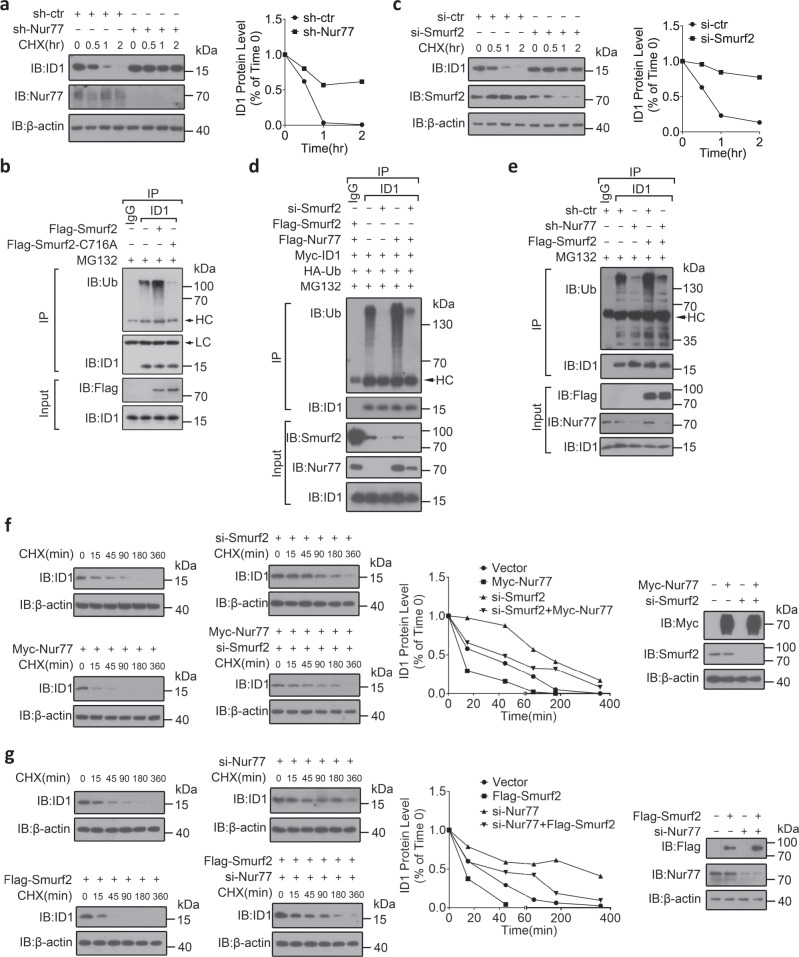
Nur77 post-translationally downregulates ID1 through mediating its association with and ubiquitylation by Smurf2. **a**, **c**, **f**, **g** SW620 cells were transfected with the indicated expression plasmids, shRNAs, or siRNAs followed by cycloheximide (CHX) (10 μM) treatment for the indicated time. Protein expressions were examined by IB. The anti-ID1 antibody signal normalized relative to the anti-actin signal is expressed as a percentage of that present at the start of the chase. si-ctr control siRNA, si-Nur77 Nur77 siRNA, si-Smurf2 Smurf2 siRNA, sh-ctr control shRNA, sh-Nur77 Nur77 shRNA, hr hour, min minute. **b**, **d**, **e** SW620 cells transfected with the indicated expression plasmids, shRNAs, or siRNAs were treated with MG132 (20 μM) for 2 h. ID1 ubiquitylation was examined by IP with anti-ID1 antibody and IB with anti-ubiquitin (Ub) antibody. LC light chain, HC heavy chain. Data represent at least two independent experiments. Source data are provided as Source Data file.

Considering the Nur77 dependence of ID1 induction by TGFβ described above, it was expected that Nur77 would upregulate basal ID1 expression. To our surprise, we found that ID1 expression in colon tissues derived from the *Nr4a1*^−/−^ (Nur77 gene knockout) mice was much higher than in colons from the wild-type mice (Supplementary Fig. 2a). Moreover, Nur77 was inversely correlated with ID1 expression in HCT116 and RKO colon cancer cells (Supplementary Fig. 2b). We, therefore, hypothesized that Nur77 destabilized ID1 protein. To exclude Smad3/Smad4-mediated transcriptional induction of ID1, we used Smad4-null SW620 and HT29 colon cancer cells (Supplementary Fig. 2c). In both cell lines, suppression and overexpression of Nur77, without affecting ID1 mRNA abundance, dramatically increased and decreased ID1 protein level, respectively (Supplementary Fig. 2d, e). We further examined the effect of Nur77 on ID1 protein turnover using cycloheximide (CHX) to block translation. Nur77 suppression greatly extended the half-life of ID1 protein (Fig. 2a). Proteasomal inhibitor MG132 but not lysosomal inhibitors alleviated Nur77-induced ID1 destabilization (Supplementary Fig. 2f). In addition, Nur77 suppression dramatically decreased ID1 ubiquitylation (Supplementary Fig. 2g). Together, these results demonstrate that Nur77 destabilizes ID1 protein through the ubiquitin-proteasome pathway.

Smurf2 has been shown to ubiquitylate and destabilize ID1^27^. In agreement, Smurf2 but not Smurf2^C716A^ strongly induced ID1 ubiquitylation, even though they both bound to ID1 (Fig. 2b and Supplementary Fig. 2h). Smurf2 suppression inhibited ID1 ubiquitylation and turnover in SW620 cells (Supplementary Fig. 2i and Fig. 2c). The ubiquitylation of ID1 was attenuated by either Smurf2 or Nur77 suppression but was completely blocked by simultaneously suppressing both (Supplementary Fig. 2i). Moreover, Nur77-enhanced ID1 ubiquitylation and turnover were blocked by Smurf2 suppression (Fig. 2d, f and Supplementary Fig. 2j), while Smurf2-mediated ID1 ubiquitylation and turnover were likewise abrogated by Nur77 suppression (Fig. 2e, g). Thus, Nur77 and Smurf2 interdependently induce ID1 ubiquitylation and degradation.

**Fig. 3:**
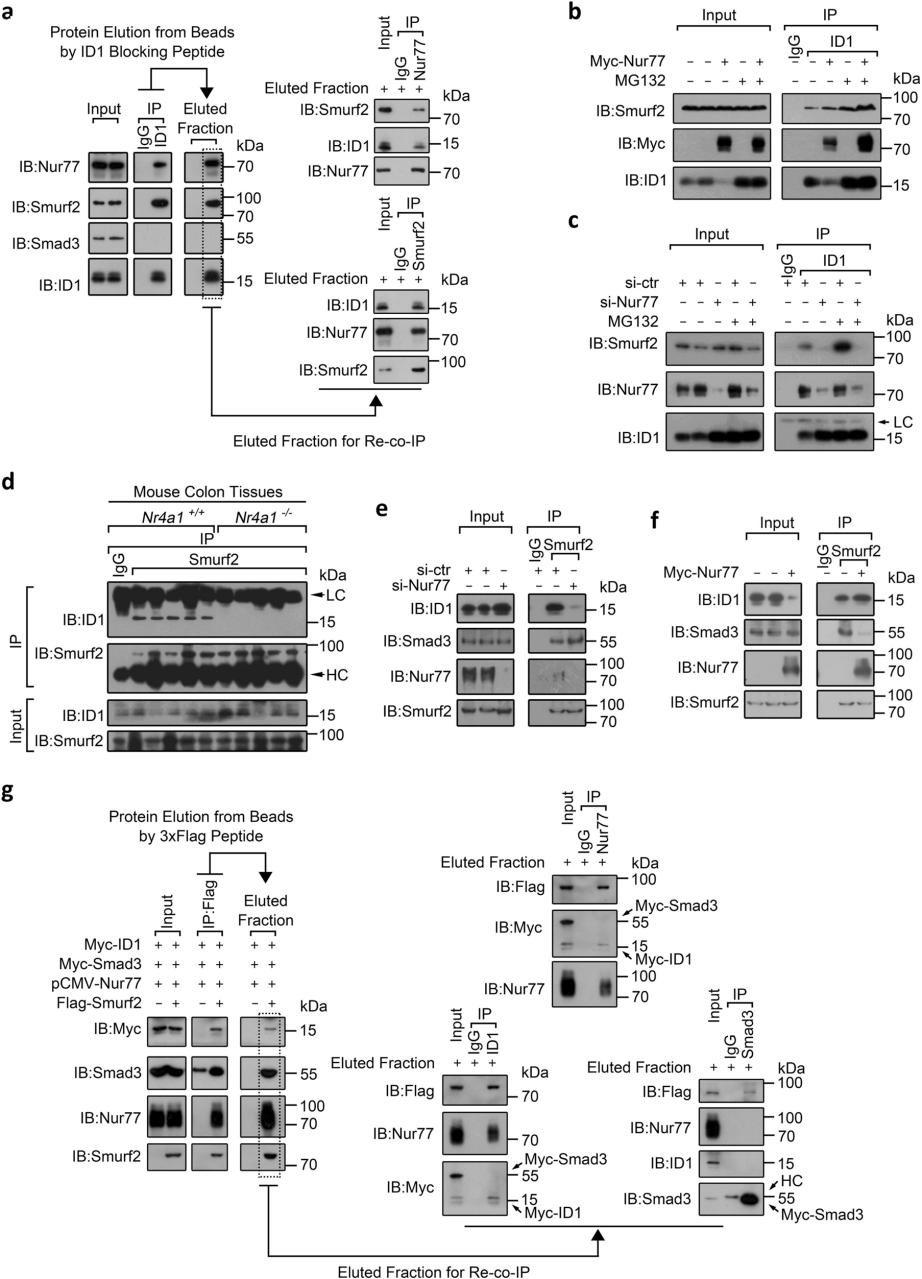
Molecular mechanisms underlying Nur77’s effects on the interactions of Smurf2 with Smad3 and ID1. **a**, **g** Re-co-immunoprecipitation assay to detect the trimeric interaction of Nur77, ID1, and Smurf2 in SW620 cells. The detailed procedure was presented in Supplementary Figure 2b and described in “Methods” section. LC light chain, HC heavy chain. **b**, **c** SW620 cells transfected with the indicated expression plasmids or siRNAs were treated with MG132 (20 μM) for 2 h. Protein interactions were examined by co-IP. si-ctr control siRNA, si-Nur77 Nur77 siRNA. **d**–**f** Protein interactions were examined in mouse colon tissues (**d**) and in SW620 cells (**e**, **f**). Data represent at least two independent experiments. Source data are provided as Source Data file.

In exploring the mechanism of the interdependent control of ID1 by Nur77 and Smurf2, we found that Nur77 interacted with both ID1 and Smurf2 (Supplementary Fig. 3a). Our re-co-immunoprecipitation (co-IP) assay demonstrated the trimeric interactions among ID1, Smurf2, and Nur77 (Fig. 3a and Supplementary Fig. 3b). Moreover, overexpression and suppression of Nur77 substantially enhanced and reduced the association of Smurf2 with ID1, respectively (Fig. 3b, c). Thus, Nur77 enhances the association of Smurf2 with ID1, followed by triggering Smurf2-mediated ID1 ubiquitylation and degradation. This is further supported by the strong association of ID1 with Smurf2 in colon tissues from wild-type but not *Nr4a1*^−/−^ mice (Fig. 3d), an observation that offers a plausible explanation for the higher ID1 levels in the colons of *Nr4a1*^−/−^ mice (Supplementary Fig. 2a).

### Molecular mechanisms underlying Nur77’s effects on the interactions of Smurf2 with Smad3 and ID1

Notably, Nur77 showed opposing effects on Smurf2-mediated mono-ubiquitylation of Smad3 and poly-ubiquitylation of ID1, likely resulting from the distinct effects of Nur77 in mediating the interactions of Smurf2 with Smad3 and ID1. Whereas Nur77 promoted Smurf2 association with ID1, it inhibited Smurf2 association with Smad3 (Fig. 3e, f). By re-co-IP assay, we found that ID1 but not Smad3 could form trimeric interactions with Nur77 and Smurf2 (Fig. 3g). In dissecting the interaction domains in Nur77, we found that the N-terminal AB region and C-terminal ligand-binding domain (LBD) of Nur77 interacted with Smurf2 and ID1, respectively (Supplementary Fig. 3c–f). Distinct binding sites in Nur77 might mediate the association of ID1 and Smurf2. We found that the AB region was also responsible for the interaction of Nur77 with Smad3 (Supplementary Fig. 3g). Thus, Smurf2 and Smad3 may compete for binding to the AB region of Nur77. Consistently, Nur77 was unable to mediate an interaction between Smurf2 and Smad3. Rather, binding of Nur77 to either Smurf2 or Smad3 may produce steric hindrance to inhibit their interaction.

### TGFβ stabilizes ID1 protein through preventing its Nur77-mediated interaction with and ubiquitylation by Smurf2

**Fig. 4:**
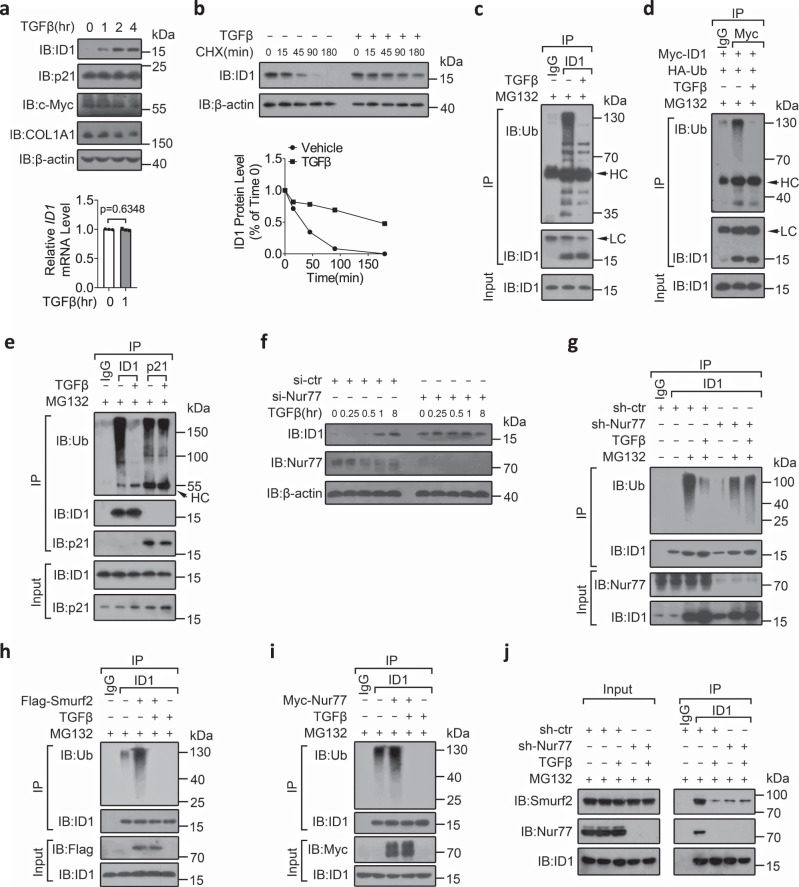
TGFβ stabilizes ID1 protein through preventing its Nur77-mediated interaction with and ubiquitylation by Smurf2. **a** SW620 cells were treated with TGFβ (10 ng/mL) for the indicated time. IB and qRT-PCR were applied to examine protein and ID1 mRNA expressions, respectively. hr hour. Two-tailed unpaired Student’s *t* test were used for statistical analysis, and data are presented as means ± SD (*n* = 3 biologically independent samples). **b** Cycloheximide (CHX) chase assay to determine ID1 turnover in the presence of TGFβ (10 ng/mL) in SW620 cells. min minute. **c**–**e**, **g**–**i** SW620 cells were transfected with the indicated expression plasmids, siRNAs, or shRNAs followed by MG132 treatment for 2 h and then TGFβ (10 ng/mL) treatment for 1 h. Protein ubiquitylation was examined by IP using the indicated antibodies and IB using anti-Ubiquitin (Ub) antibody. LC light chain, HC heavy chain, sh-ctr control shRNA, sh-Nur77 Nur77 shRNA. **f** SW620 cells were transfected with siRNAs before treatment with TGFβ (10 ng/mL) for the indicated times. Protein expressions were examined by IB. si-ctr control siRNA, si-Nur77 Nur77 siRNA. **j** SW620 cells transfected with the indicated shRNAs were treated with MG132 for 2 h and then TGFβ (10 ng/mL) for 1 h. Co-IP was applied to detect protein interactions. Data represent at least two independent experiments. Source data are provided as Source Data file.

TGFβ was deemed unlikely to induce ID1 expression in SW620 cells because of its inability to induce ID1 mRNA due to Smad4 deficiency (Supplementary Fig. 2c and Fig. 4a). Nonetheless, we found that TGFβ markedly and time-dependently increased the protein level of ID1 but not p21, c-Myc, or COL1A1, which are encoded by three other TGFβ target genes^38–40^ (Fig. 4a). This finding implied that TGFβ selectively stabilized ID1 protein. Indeed, TGFβ potently extended ID1 half-life in SW620 cells (Fig. 4b). Furthermore, TGFβ strongly inhibited the ubiquitylation of endogenous and exogenous ID1 but not p21 or c-Myc (Fig. 4c–e and Supplementary Fig. 4a). TGFβ-induced inhibition of ID1 ubiquitylation depended on TβRI activation because ca-TβRI, a constitutively active form of TβRI^36^, dose-dependently inhibited ID1 ubiquitylation and SB431542 blocked the effect of TGFβ on ID1 ubiquitylation (Supplementary Fig. 4b, c). However, this effect did not require TGFβ-stimulated gene transcription because CHX had no effect on TGFβ-induced inhibition of ID1 ubiquitylation (Supplementary Fig. 4d). Together, these results reveal a previously unrecognized role of TGFβ in increasing ID1 protein through modulation of its ubiquitylation and stability.

The effects of TGFβ on induction of ID1 expression and inhibition of ID1 ubiquitylation were largely abolished when Nur77 was knocked down (Fig. 4f, g and Supplementary Fig. 4e). Interestingly, TGFβ blocked Smurf2-mediated ID1 ubiquitylation (Fig. 4h), apparently by disrupting the interaction of Smurf2 with ID1 (Supplementary Fig. 4f). TGFβ also blocked Nur77-induced ID1 ubiquitylation and Nur77-dependent Smurf2–ID1 association (Fig. 4i, j and Supplementary Fig. 4g). Together, these results demonstrate that TGFβ inhibits Smurf2-mediated ID1 ubiquitylation by blocking Nur77-induced Smurf2 association with ID1.

### TGFβ converts Nur77 role in regulating ID1 expression

**Fig. 5:**
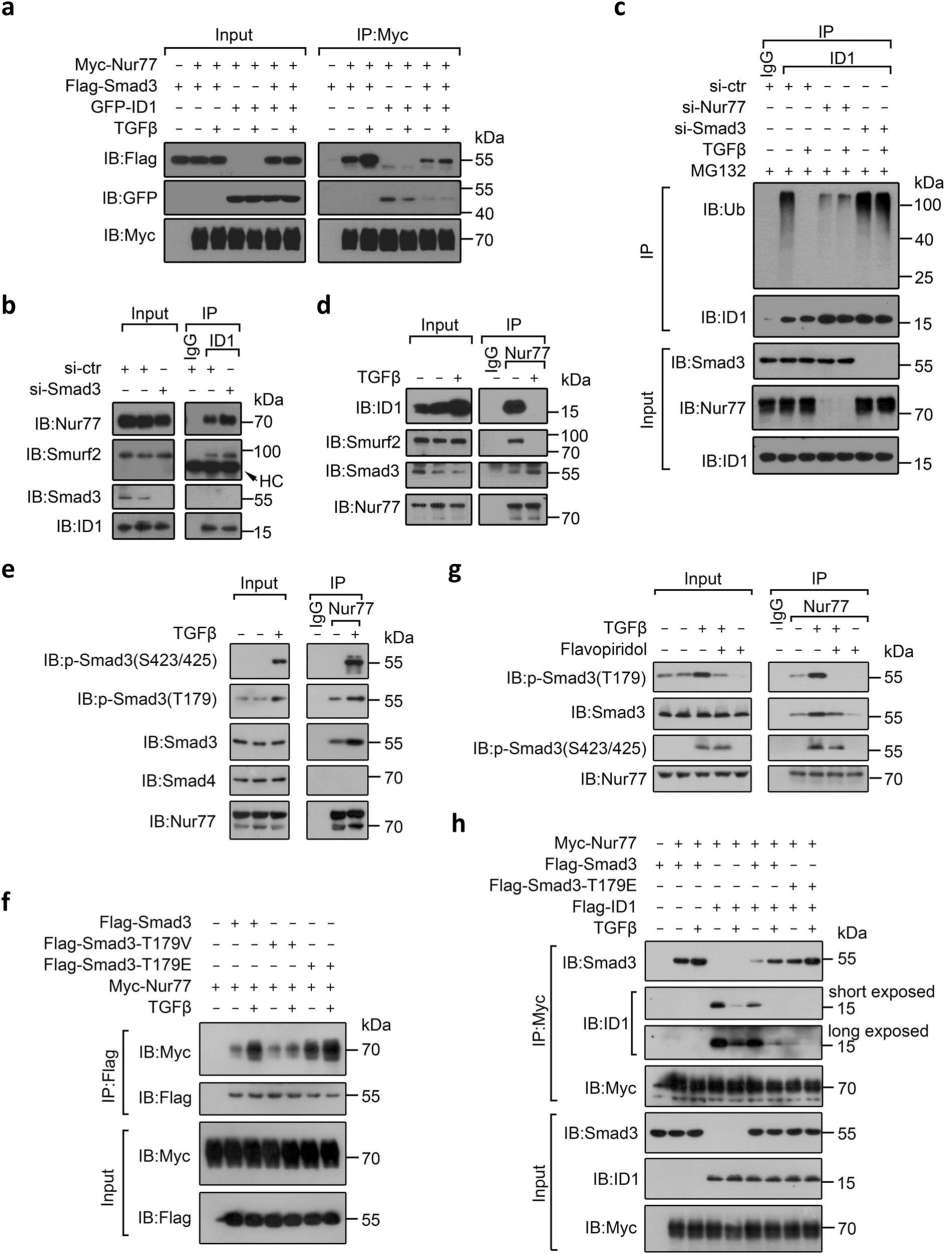
TGFβ converts Nur77 role in regulating ID1 expression. **a**, **b**, **d**, **f**, **h** HCT116 cells transfected with the indicated expression plasmids or siRNA were treated with TGFβ (10 ng/mL) for 1 h. Protein interactions were examined by co-IP. si-ctr control siRNA, si-Smad3 Smad3 siRNA. **c** HCT116 cells transfected with the indicated siRNAs were treated with MG132 for 2 h prior to TGFβ (10 ng/mL) treatment for 1 h. ID1 ubiquitylation was examined. si-Nur77 Nur77 siRNA. **e**, **g** LS174T cells were pretreated with flavopiridol (0.25 μM) for 1 h (**g**), and stimulated by TGFβ (10 ng/mL) for 1 h. Protein interactions were examined by co-IP. Data represent at least two independent experiments. Source data are provided as Source Data file.

The above studies suggested a model in which ID1 and Smad3 compete for Nur77 binding. Indeed, Smad3 and ID1 mutually inhibited their interaction with Nur77 in HCT116 cells (Fig. 5a). Smad3 suppression enhanced ID1 interaction with Nur77 and Smurf2 (Fig. 5b), and consequently increased ID1 ubiquitylation (Fig. 5c). Notably, TGFβ inhibited the interaction of ID1, but enhanced the interaction of Smad3 with Nur77 (Fig. 5a, d). This, in turn, resulted in TGFβ inhibiting Nur77-mediated ID1 degradation, but enhancing Nur77-mediated ID1 mRNA transcription. Although Nur77 and Smad3 exerted opposite effects on ID1 ubiquitylation, they were both required for TGFβ-induced inhibition of ID1 ubiquitylation (Fig. 5c). Thus, TGFβ enhanced Smad3, but inhibited ID1 interaction with Nur77, resulting in converting Nur77 from a negative post-translational regulator to a positive transcriptional regulator of ID1 expression.

Although Nur77 interacted with ID1 via its LBD, the inhibitory effect of TGFβ on the interaction of Nur77 and ID1 required Nur77’s AB region that was responsible for the binding of Nur77 to Smad3 (Supplementary Fig. 5a), implying that TGFβ-induced conversion of Nur77 involved Smad3. TβRI-mediated phosphorylation of Smad3 on Ser423/425 is essential for TGFβ/Smad3 signal transduction^41^. We first hypothesized that the phosphorylated Smad3 (p-Smad3^S423/425^) had increased affinity for Nur77, thus competing more readily with ID1. Although p-Smad3^S423/425^ interacted with Nur77 (Supplementary Fig. 5b), there was no significant difference in Nur77’s interaction with Smad3, the phosphorylation-defective mutant Smad3^S423/425A^, or the phosphorylation-mimic mutant Smad3^S423/425D^. Moreover, their binding to Nur77 was equally induced by TGFβ (Supplementary Fig. 5c). Thus, Ser423/425 phosphorylation is not essential for TGFβ-induced interaction of Smad3 with Nur77.

By analyzing Smad3 deletion mutants, we found that its MH2 but not the MH1 domain mediated binding to Nur77 (Supplementary Fig. 5d, e). The ΔMH1 mutant we created retained the linker region and the MH2 domain, whereas the MH2 mutant only contained the MH2 domain (Supplementary Fig. 5d). Although the MH2 mutant showed higher Nur77 binding than the ΔMH1 mutant, the interaction of Nur77 with the ΔMH1 but not the MH2 mutant was enhanced by TGFβ (Supplementary Fig. 5f), implying that the linker region contained the TGFβ-responsive elements regulating the Nur77–Smad3 interaction. TGFβ is known to induce CDK2/4-catalyzed phosphorylation of Smad3 at Thr179 within the linker region^42,43^. TGFβ-stimulated Thr179 phosphorylation was also observed in LS174T cells, and the T179–p-Smad3 interacted with Nur77 (Fig. 5e). Importantly, the phosphorylation-mimic mutant Smad3^T179E^ exhibited stronger Nur77 binding than wild-type Smad3 or phosphorylation-defective mutant Smad3^T179V^ (Fig. 5f). Nur77 affinity to Smad3^T179E^ was comparable to that of TGFβ-stimulated Smad3. In addition, the CDK inhibitor flavopiridol, which inhibited the phosphorylation of Smad3 at Thr179, reduced TGFβ-induced interaction of Nur77 with Smad3 (Fig. 5g). Moreover, Smad3^T179E^ was more effective than Smad3 in inhibiting the interaction of ID1 with Nur77 (Fig. 5h). Thus, TGFβ promotes Smad3 phosphorylation at Thr179 to enhance its interaction with Nur77 and thereby reduce Nur77’s interaction with ID1, resulting in Nur77’s role conversion.

The fact that TGFβ still enhanced the interaction of Nur77 and Smad3^T179E^ suggested the existence of other mechanisms underlying TGFβ-induced Nur77 conversion (Fig. 5f, h). That knockdown of Smad3 was not able to completely block the inhibitory effect of TGFβ on the interaction of ID1 and Nur77 suggested the existence of additional Smad3-unrelated mechanisms, which may involve TGFβ-induced Nur77 modifications (Supplementary Fig. 5g). Thus, TGFβ appears to engage multiple mechanisms to convert Nur77 role, which remains to be explored.

### Pathophysiological relevance of the TGFβ/Nur77/ID1 axis in colon cancer

**Fig. 6:**
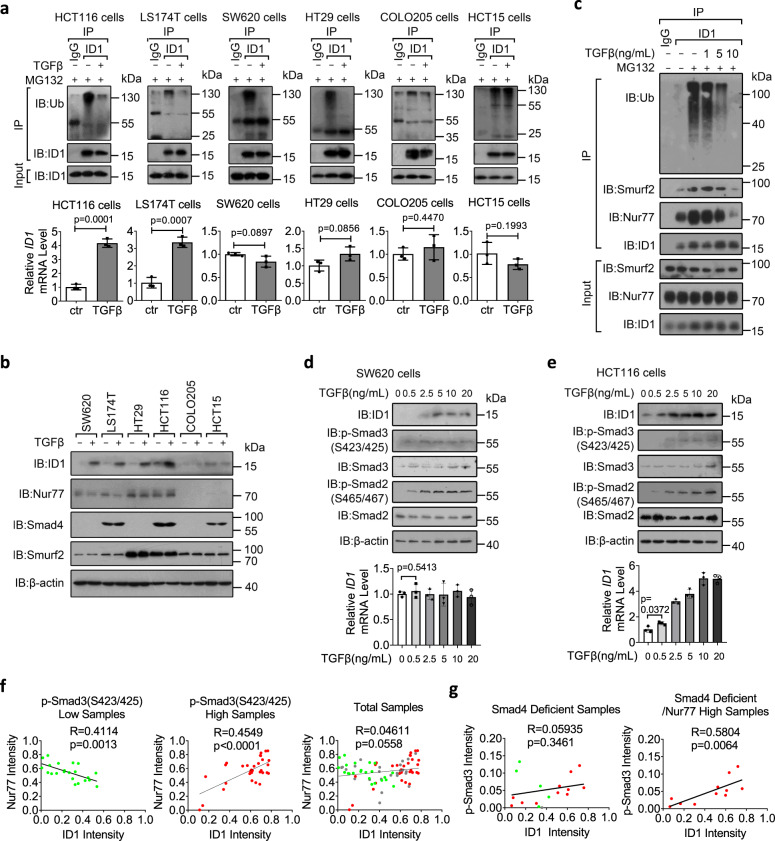
Pathophysiological relevance of the TGFβ/Nur77/ID1 axis in colon cancer. **a** Cells pretreated with MG132 (20 μM) for 2 h were then treated with TGFβ (10 ng/mL) for 1 h. ID1 ubiquitylation and mRNA expression were examined by co-immunoprecipitation and qRT-PCR, respectively. Ub ubiquitin. Two-tailed unpaired Student’s *t* test was used for statistical analysis, and data are presented as means ± SD (*n* = 3 biologically independent samples). **b** Cells were treated with TGFβ (10 ng/mL) for 1 h and protein expression was examined by immunoblotting using the indicated antibodies. **c** SW620 cells were treated with MG132 for 2 h and then with TGFβ at the indicated doses for 1 h. ID1 ubiquitylation and protein interactions were examined. **d**, **e** SW620 (**d**) and HCT116 (**e**) cells were treated with TGFβ at the indicated doses for 1 h. IB and qRT-PCR were applied to examine protein and ID1 mRNA expressions, respectively. The phosphorylation of Smad2 and Smad3 were detected using anti-p-Smad2(S465/467) and anti-p-Smad3(S423/425) antibodies, respectively. Two-tailed unpaired Student’s *t* test was used for statistical analysis, and data are presented as means ± SD (*n* = 3 biologically independent samples). **f**, **g** Immunohistochemistry analysis of clinical colon cancer samples showing correlations of ID1 expression with Nur77 expression and TGFβ signal activity. The green (*n* = 22) and red (*n* = 33) dots represented tissue samples with low and high TGFβ signal activity, respectively (**f**). The green (*n* = 5) and red (*n* = 11) dots represented tissue samples with low and high Nur77 expression, respectively (**g**). Two-tailed correlation analysis was used to indicate correlation (assume data were sampled from Gaussian population). Data represent at least two independent experiments. Source data are provided as Source Data file.

To address the relevance of the above molecular events for colon cancer, we studied several Smad4-proficient and Smad4-deficient colon cancer cell lines. As a critical transducer in canonical TGFβ signaling, Smad4 was essential for the induction of ID1 mRNA by TGFβ, revealed by the failure of TGFβ to upregulate ID1 mRNA in SW620, HT29, and COLO205 cells lacking Smad4 expression (Fig. 6a, b). However, Smad4 was dispensable for TGFβ to regulate ID1 ubiquitylation. First, TGFβ inhibited ID1 ubiquitylation in both Smad4-proficient HCT116 and LS174T cells and Smad4-deficient SW620 and HT29 cells (Fig. 6a, b). Second, Smad4 knockdown did not affect the ability of TGFβ in regulating ID1 ubiquitylation in HCT116 cells (Supplementary Fig. 6a). Third, Smad4 did not affect TGFβ regulation of the interactions of Nur77 with ID1, Smad3, or Smurf2 (Supplementary Fig. 6b). Notably, the potent effects of TGFβ on inhibiting ID1 ubiquitylation and inducing ID1 protein expression were found in HCT116, LS174T, SW620, and HT29 cells that expressed high levels of Nur77, but not in COLO205 and HCT15 cells with low Nur77 (Fig. 6a, b). This cell line selectivity corroborated the essential role of Nur77 in ID1 induction by TGFβ.

As shown in Fig. 6c, d, TGFβ dose-dependently disrupted ID1/Nur77/Smurf2 complex coincident with inhibition of ID1 ubiquitylation and induction of ID1 protein expression in SW620 cells. Notably, there existed a threshold concentration of TGFβ (≥5 ng/mL) required for its effective inhibition of complex formation and ID1 ubiquitylation as well as stabilization of ID1 protein in SW620 cells (Fig. 6c, d). Interestingly, TGFβ at lower concentration (≤0.5 ng/mL) induced Smad2/3 phosphorylation as well as ID1 mRNA and protein expression in HCT116 cells (Fig. 6e), corresponding to the lower concentration of TGFβ required for inhibiting Smad3 ubiquitylation (Supplementary Fig. 1k). These results indicate that a higher concentration of TGFβ is required for stabilizing ID1 protein than for inducing *ID1* gene transcription in colon cancer cells.

To further explore the pathophysiological relevance of the mechanisms discovered in vitro, we analyzed the correlation of Nur77 and ID1 expression in tissue samples from colon cancer patients. In the light of the TGFβ-induced role conversion of Nur77 in ID1 expression, we divided the tissue samples into low and high TGFβ signal groups based on the extent of Smad3 phosphorylation (Supplementary Fig. 6c–e). In the low TGFβ signal group, Nur77 was inversely correlated with ID1 expression (Fig. 6f), reflecting the role of Nur77 in promoting ID1 degradation. Conversely, in the high TGFβ signal group, Nur77 was positively correlated with ID1 expression, reflecting that a high TGFβ signal converted Nur77 from a negative to a positive regulator of ID1 expression. It was thus not surprising that no correlation between Nur77 and ID1 levels was found if the tissue samples were not grouped by TGFβ signal activity (Fig. 6f).

We showed above that TGFβ upregulated ID1 expression in Smad4-deficient cells through stabilizing ID1 protein. However, we did not observe a correlation between TGFβ signal activity and ID1 expression in Smad4-deficient clinical colon cancer samples (Fig. 6g and Supplementary Fig. 6c). This was reminiscent of the Nur77-dependent effect of TGFβ on ID1 stabilization. Indeed, ID1 expression and TGFβ signal activity were positively correlated in Smad4-deficient colon cancer tissues with relatively high Nur77 expression (Fig. 6g and Supplementary Fig. 6c, d, f).

### Involvement of the TGFβ/Nur77/ID1 axis in colon cancer stemness and metastasis

**Fig. 7:**
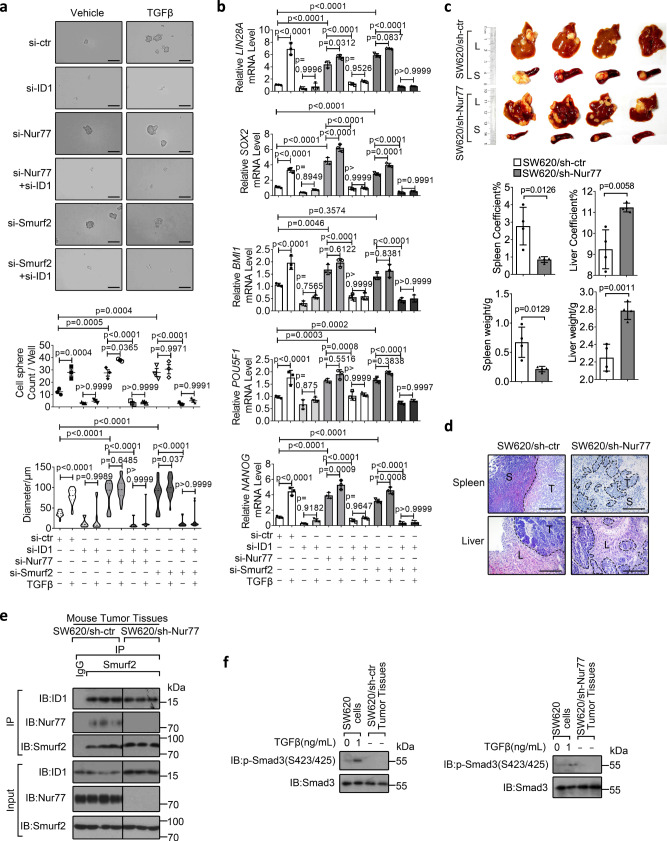
Involvement of the TGFβ/Nur77/ID1 axis in colon cancer stemness and metastasis. **a**, **b** LS174T cells transfected with the indicated siRNAs were cultured for 7 days. Cell spheres were observed by microscope. Representative images were shown, and sphere numbers and diameters were counted and measured (**a**). Scale bars, 100 μm. The expression of stemness markers was determined by qRT-PCR (**b**). si-ctr control siRNA, si-ID1 ID1 siRNA, si-Nur77 Nur77 siRNA, si-Smurf2 Smurf2 siRNA. Two-way ANOVA followed by Tukey’s multiple comparisons test was used for statistical analysis, and data are presented as means ± SD. (**a**, top graph, *n* = 3 biologically independent samples; **a**, bottom graph, *n* = 37, 60, 9, 11, 35, 33, 7, 9, 32, 25, 6, 14, respectively; **b**, *n* = 5 biologically independent samples). **c**–**f** SW620/sh-ctr and SW620/sh-Nur77 cells were injected into spleens of nude mice. Mice were reared for 28 days and then sacrificed for analysis of tumor formation (**c**), tumor-bearing spleen and liver weight and coefficient (**c**), hematoxylin and eosin staining of tumor tissues (**d**), protein interactions and expressions in spleen tumors (**e**), and Smad3 phosphorylation status in spleen tumors comparing to in vitro SW620 cells treated with TGFβ (**f**). The white areas indicate tumors formed in spleens and livers (**c**). Tumor tissues are separated from normal tissues by dotted lines (**d**). S spleen, L liver, T tumor. Scale bars, 100 μm. Two-tailed unpaired Student’s *t* test was used for statistical analysis, and data are presented as means ± SD (*n* = 4 mice per group). Data represent at least two independent experiments. Source data are provided as Source Data file.

ID1 is a pivotal factor fostering cancer cell stemness^44^. Accordingly, we used in vitro three-dimensional (3D) cell sphere assay to analyze the effect of Nur77 and TGFβ on regulating ID1-dependent stemness of colon cancer cells^19,45,46^. LS174T and SW620 cells formed small spheres with diameters of 40 μm in 3D cultures (Fig. 7a and Supplementary Fig. 7a). Sphere numbers and sizes as well as the expression of stemness markers *NANOG*, *SOX2*, *BMI1*, *LIN28A*, and *POU5F1* were greatly increased by either Nur77 suppression or TGFβ treatment, which was abolished by ID1 suppression (Fig. 7a, b and Supplementary Fig. 7a). This indicates that Nur77 and TGFβ act, respectively, as a negative and positive regulator of cell stemness in an ID1-dependent manner, which fits with our observation that Nur77 and TGFβ downregulated and upregulated ID1 expression, respectively. This is also consistent with previous findings that ID1 is a positive regulator in colon cancer cell stemness^47–49^. The combination of Nur77 suppression and TGFβ treatment did not produce strong synergistic effects on sphere formation and stemness marker expression (Fig. 7a, b and Supplementary Fig. 7a), in agreement with Nur77 mediating the effect of TGFβ on ID1 induction. Smurf2 exhibited similar effects as Nur77 on sphere formation and stemness marker expression, likely reflecting their interdependence on ID1 downregulation (Fig. 7a, b).

Considering the critical role of ID1 in colon cancer metastasis, we explored the significance of the above mechanisms in hepatic metastasis of colon cancer. A hepatic metastasis model was generated in nude mice using a splenic injection of SW620/shRNA-control (sh-ctr) and SW620/shRNA-Nur77 (sh-Nur77) stable cell lines, and tumors formed in spleens and livers were examined (Fig. 7c). While tumors formed by sh-ctr cells were sharply demarcated from surrounding normal tissues, sh-Nur77 tumors were more invasive with irregular linings between normal and tumor tissues (Fig. 7d). Consistently, sh-Nur77 tumors had higher liver metastatic potential than sh-ctr tumors (Fig. 7c). Sh-Nur77 tumors showed higher ID1 expression (Fig. 7e), which may be responsible for hepatic metastasis of colon cancer cells^50^. In addition, Nur77 positively regulated the interaction of Smurf2 and ID1 in splenic tumor tissues (Fig. 7e). We also found that TGFβ signal was relatively low in these tumors (Fig. 7f and Supplementary Fig. 7b). Together, these results indicate that Nur77 inversely correlates with ID1 expression and colon cancer metastasis in the environment of low TGFβ signaling in vivo.

### Involvement of the TGFβ/Nur77/ID1 axis in colon cancer resistance to oxaliplatin

**Fig. 8:**
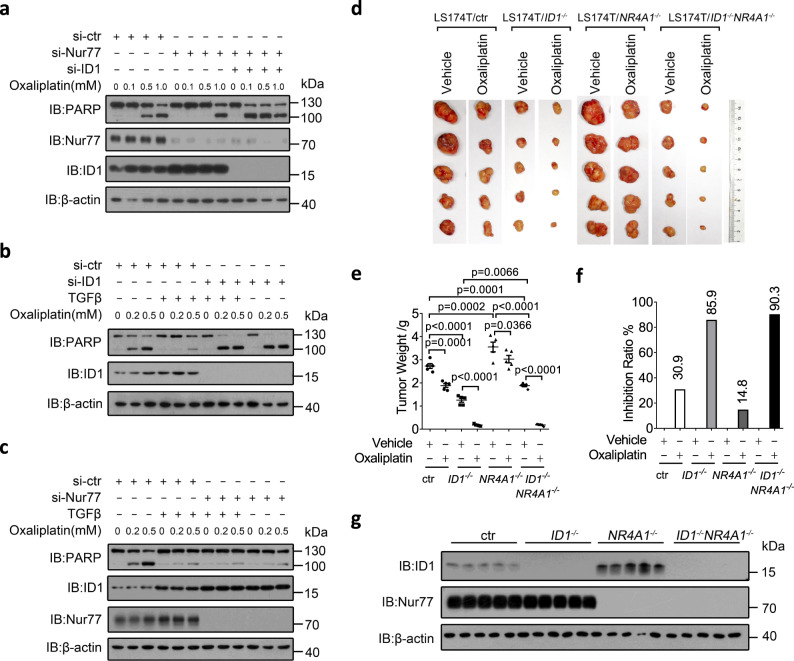
Involvement of the TGFβ/Nur77/ID1 axis in colon cancer resistance to oxaliplatin. **a**–**c** LS174T cells transfected with siRNAs were treated with the indicated doses of oxaliplatin in the presence or absence of TGFβ (10 ng/mL) for 12 h. Protein expressions were examined by IB. si-ctr control siRNA, si-ID1 ID1 siRNA, si-Nur77 Nur77 siRNA. **d**–**g** The indicated LS174T cell lines were inoculated subcutaneously into flanks of nude mice. After 10 days, mice were intraperitoneally injected with oxaliplatin (5 mg/kg) daily. After 12 days, mice were sacrificed for analysis of dissected tumors (**d**), tumor weights (**e**), tumor inhibition ratios (**f**), and protein expressions in tumors (**g**). Two-way ANOVA followed by Tukey’s multiple comparisons test was used for statistical analysis, and data are presented as means ± SD (*n* = 5 mice per group). Data represent at least two independent experiments. Source data are provided as Source Data file.

ID1 expression contributes to the resistance of colon cancer to the chemotherapeutic drug oxaliplatin through upregulating cancer cell self-renewal capacity^19^. Consistently, we found that ID1 expression was positively correlated with LS174T cell resistance to oxaliplatin-induced apoptosis (Supplementary Fig. 8a, b). In contrast, Nur77 expression was inversely correlated with cell resistance to oxaliplatin (Supplementary Fig. 8c, d). Increased oxaliplatin resistance resulting from Nur77 suppression was accompanied by increased ID1 expression and abolished by ID1 suppression (Fig. 8a). Thus, the inverse correlation between Nur77 and oxaliplatin resistance appears to result from the inverse relationship between Nur77 and ID1 expression. TGFβ treatment also increased LS174T cell resistance to oxaliplatin, which was abolished by ID1 suppression (Fig. 8b). TGFβ-induced oxaliplatin resistance and ID1 expression were both eliminated by Nur77 suppression (Fig. 8c). Thus, the effect of TGFβ on increasing colon cancer resistance to oxaliplatin appears to arise from its Nur77-dependent induction of ID1 expression.

These mechanisms were further explored in a mouse xenograft study. Nude mice were subcutaneously inoculated with LS174T-knockout cell lines (control, *ID1*^−/−^, *NR4A1*^−/−^ (Nur77 gene knockout) and *ID1*^−/−^ *NR4A1*^−/−^) and then treated with oxaliplatin. We found that *ID1* knockout inhibited tumor growth (Fig. 8d, e, g), signifying the pro-tumorigenic effect of ID1. In contrast, *NR4A1* knockout promoted tumor growth coincident with increased ID1 expression in tumor tissues (Fig. 8d, e, g). Tumor promotion resulting from *NR4A1* knockout was abolished when *ID1* was knocked out simultaneously, whereas the tumor inhibitory effect of *ID1* knockout was not significantly affected by *NR4A1* knockout (Fig. 8d, e, g). This places ID1 downstream of Nur77 in tumor growth control. Consistent with the inverse correlation of Nur77 and ID1 expression was our finding that TGFβ signal was low in these tumor tissues (Supplementary Fig. 8e).

Treatment of mice with oxaliplatin modestly inhibited the growth of control tumors (Fig. 8d–f and Supplementary Fig. 8f). *ID1* knockout sensitized tumors to oxaliplatin (Fig. 8d–g and Supplementary Fig. 8f), again demonstrating the contribution of ID1 to oxaliplatin resistance. On the contrary, *NR4A1* knockout rendered tumors more resistant to oxaliplatin (Fig. 8d–g and Supplementary Fig. 8f). This demonstrated again that Nur77 negatively correlates with oxaliplatin resistance likely due to its reverse relationship with ID1 expression. Compared to *NR4A1*^−/−^ tumors, oxaliplatin exhibited a more potent inhibitory effect in *ID1*^−/−^*NR4A1*^−/−^ tumors (Fig. 8d–g and Supplementary Fig. 8f), indicating that ID1 upregulation due to *NR4A1* knockout caused oxaliplatin resistance in *NR4A1*^−/−^ tumors. No significant difference in the sensitivity to oxaliplatin was observed between *ID1*^−/−^ and *ID1*^−/−^*NR4A1*^−/−^ tumors (Fig. 8d–g and Supplementary Fig. 8f), indicating that ID1 mediated the effect of Nur77 and Nur77 acted upstream of ID1 in tumor growth regulation.

## Discussion

ID1, a potent promoter of cancer growth, metastasis, and stemness^23,35,51–54^, represents an attractive drug target for cancer treatment. However, direct targeting of ID1 protein by small molecules may not be feasible because of its small size and the absence of proper sites for small-molecule binding^54^. Alternative approaches such as designing small molecules targeting key proteins that modulate ID1 expression may indirectly decrease its level, thus producing desirable anticancer effects^55,56^.

**Fig. 9:**
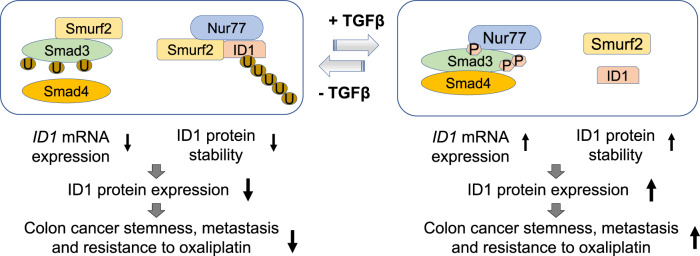
A working model. The crosstalk of Nur77 and TGFβ on dual regulations of ID1 expression and the implications in colon cancer progression and oxaliplatin resistance. U ubiquitin, P phosphate.

ID1 is a key TGFβ target gene that mediates the oncogenic effects of TGFβ^22,25,57,58^. It is widely thought that TGFβ signaling increases ID1 abundance at the mRNA level^57^. Here, we show that TGFβ also upregulates ID1 expression post-translationally through inhibiting its ubiquitin-mediated degradation, providing an unidentified mechanism for TGFβ-induced upregulation of ID1. This finding signifies the role of TGFβ in cancer promotion in two aspects. First, TGFβ can efficiently increase ID1 expression in cancer cells through both mRNA induction and protein stabilization. Secondly, TGFβ can actively exert its oncogenic effect in Smad4-deficient cancers through stabilizing ID1 protein. Thus, Smad4-deficient colon cancer cells not only evade TGFβ-induced growth inhibition through Smad4 deletion but also actively respond to TGFβ-induced tumor progression at least through ID1 stabilization, thus reflecting their high malignancy. Thus, our results invoke a model according to which TGFβ signaling exerts dual regulations of specific protein expression (Fig. 9), a principle that may apply to other signaling pathways as well.

Significantly, we found that higher concentrations of TGFβ are required for ID1 stabilization than for ID1 mRNA induction. In normal tissues with low TGFβ levels, TGFβ would moderately upregulate ID1 expression only via transcriptional induction^4^. In tumors, however, high levels of TGFβ will lead to ID1 overexpression resulting from not only the increased ID1 mRNA induction but also the induced ID1 protein stabilization (Fig. 9). The effect of TGFβ on ID1 stabilization may be more significant in tumor development. TGFβ can stabilize not only basal and its own-induced ID1 but also ID1 induced by other signals, thus serving as a mechanism for the interplay between TGFβ and other signals for promoting cancer progression.

We also discovered that Nur77 has dual activity in regulating ID1 expression in colon cancer, a finding that likely reflects a combination of genomic and non-genomic mechanisms of Nur77. When TGFβ signal is low or absent, Nur77 acts as a negative regulator of ID1 by enhancing Smurf2-mediated ID1 ubiquitylation and degradation. However, TGFβ can convert Nur77 from a negative to a positive regulator. This likely occurs in cancers when a high TGFβ signal disrupts Nur77-mediated ID1 interaction with and ubiquitylation by Smurf2. Meanwhile, TGFβ induces Nur77 interacting with Smad3 to enhance *ID1* gene transcription. Thus, it is Nur77 that mediates the dual modes of TGFβ in regulating ID1 expression, while it is TGFβ that converts the role of Nur77 in regulating ID1 expression (Fig. 9).

The role of Nur77 in cancer development is ambivalent as it has both cancer-promoting and cancer-suppressing activities^59–64^. While these conflicting roles may be attributed to cell- and tissue-specific differences, Nur77 may function in opposite ways even within one cancer type depending on the environmental context. Specifically, we found that the function of Nur77 in colon cancer is defined by its effects on ID1 expression and is switched by the strength of TGFβ signal. In an environment of low TGFβ signal, Nur77 acts as a tumor suppressor by reducing ID1 expression. In high TGFβ signal conditions, Nur77 acts as a tumor promoter by amplifying the effect of TGFβ on ID1 induction (Fig. 9).

The integrated genomic and non-genomic actions of Nur77 in ID1 regulation indicate that Nur77 is a critical regulator of ID1 expression and a potential drug target for downregulating ID1 expression. Importantly, the functions of Nur77 are tightly regulated by its small molecular ligands. Future research will evaluate the potential of Nur77 ligands in downregulating ID1 expression, thereby providing clinical significance in colon cancer treatment.

In conclusion, the mechanistic principles emerging from our study highlight the tightly integrated duality of Nur77 and TGFβ signaling in regulating ID1 expression as well as its implications in colon cancer development and treatment.

## Methods

### Mice

All animal experiments were performed in accordance with the National Institutes of Health guide for the care and laboratory animals and with the approval of the Animal Care and Use Committee of Xiamen University. The *Nr4a1* gene knockout mice (*Nr4a1* C57BL/6J, Stock No.: 006187) and control mice (C57BL/6J, Stock No.: 000664) were purchased from the Jackson Laboratory (Bar Harbor, Maine, USA). BALB/C nude mice were purchased from the Charles River (Peking, China). All experiments were performed on female cohorts at 2 months of age. All mice were housed in pathogen-free facilities, in a 12-h light/dark cycle with temperatures of 21–23 °C and 50–55% humidity. Mice were housed together when possible in ventilated cages, with chow and water supply ad libitum. The mice were monitored daily for signs of health and distress.

### Cell lines

HCT116 (CCL-247), LS174T (CL-188), RKO (CRL-2577), SW620 (CCL-227), HT29 (HTB-38), COLO205 (CCL-222), and HCT15 (CCL-225) colon cancer cells and HEK293T (CRL-3216) kidney cells were obtained from American Type Culture Collection. SW620, LS174T, and HEK293T cells were maintained in Dulbecco’s modified Eagle’s medium (DMEM) supplemented with 10% (vol/vol) fetal bovine serum (FBS) and 100 U/mL penicillin–streptomycin. HCT116, RKO, COLO205, HCT15, and HT29 cells were maintained in RPMI-1640 medium supplemented with 10% (vol/vol) FBS and 100 U/mL penicillin–streptomycin. All cells used in this study were cultivated at 37 °C with 5% CO_2_.

### Antibodies

For immunoblotting (IB), COL1A1 (#8784), c-Myc (#789), ID1 (#133104 and #488), Smurf2 (#393848), Smad4 (#7966), Myc (#40), HA (#7392), and ubiquitin (#8017 and #9133) antibodies were purchased from Santa Cruz Biotechnology and used at 1:1000 dilution. Nur77 (#3960), Smad2 (#5339), p-Smad2 (S465/467) (#3108), Smad3 (#9523), p-Smad3 (S423/425) (#9520), p21 Waf1/Cip1 (#2947), ubiquitin (#3936), and PARP (#9542) antibodies were purchased from Cell Signaling Technology and used at 1:1000 dilution. p-Smad3 (T179) (#ab74062, dilution 1:1000) was from Abcam. Smad7 (#MAB2029, dilution 1:1000) antibody was from R&D Systems. β-Actin (#A5441, dilution 1:10,000) and Flag (#F1804, dilution 1:10,000) antibodies were from Sigma-Aldrich. The goat anti-mouse IgG F(ab′)2 secondary antibody (#31436, dilution 1:10,000) and the goat anti-rabbit IgG F(ab′)2 secondary antibody (#31461, dilution 1:10,000) were from Pierce Chemical. EasyBlot anti-mouse IgG (horseradish peroxidase (HRP)) (#221667-01, dilution 1:1000) and EasyBlot anti-Rabbit IgG (HRP) (#221666-01, dilution 1:1000) were from GeneTex. For immunoprecipitation, c-Myc (#789), ID1 (#133104), Smurf2 (#393848), Myc (#40), and HA (#7392) antibodies were obtained from Santa Cruz Biotechnology and used at 1:50 dilution. Nur77 (#3960), Smad3 (#9523) and p21 Waf1/Cip1 (#2947) antibodies were obtained from Cell Signaling Technology and used at 1:100 dilution. Flag (#F1804, dilution 1:100) was from Sigma-Aldrich. For chromatin immunoprecipitation (ChIP), Smad3 (#208182, dilution 1:100) antibody was from Abcam. For immunohistochemistry staining, Nur77 (#3960, Cell Signaling Technology), p-Smad3 (S423/425) (#52903, Abcam), ID1 (#133104, Santa Cruz Biotechnology), and Smad4 (#7966, Santa Cruz Biotechnology) antibodies were used at 1:100 dilution.

### Chemical compounds

The information for commercially available chemical compounds and anticancer drugs is provided in Supplementary Table 1.

### Colon cancer tissue array

Colon cancer tissue arrays (*n* = 80) were purchased from Superchip company (Shanghai, China). Immunohistochemistry analysis of serial paraffin-embedded sections (HColA160CS01-M018, HColA160CS01-M019, HColA160CS01-M020, and HColA160CS01-M021) was conducted for examining the expressions of ID1, Nur77, Smad4, and p-Smad3 (S423/425).

### Plasmids

ID1, Smurf2, and Smads complementary cDNAs were inserted into vectors (pCMV-Myc and pFlag-CMV-2); pSuper-Nur77 plasmid was achieved by inserting TCTGGTTCCCTGGACGTTA sequence with loop sequence (TTCAAGAG) into pSuper-H1 vector. Specific details will be provided on request.

### Gene silencing by siRNA

Briefly, siRNA was transfected into cells using Lipofectamine 2000 transfection reagent (Thermo Fisher, Cat# 11668019) according to the manufacturer’s protocols. Nontargeting siRNA served as a negative control. The specific siRNA information can be found in Supplementary Table 2.

### CAGA-luciferase reporter assay

HCT116 or HEK293T cells were seeded into a 48-well plate (1 × 10^5^ cells/well) for 12 h. Cells were transfected with the CAGA-luciferase reporter (50 ng/well), Renilla (10 ng/well), and/or nuclear receptor plasmids (50 ng/well) using Lipofectamine 2000 transfection reagent (Thermo Fisher). After 24 h, cells were treated with agents as indicated in figure legends for 12 h. Medium-discarded cells were lysed by Passive Lysis Buffer (Promega, Cat# E1960), and the activities of firefly and Renilla luciferase were measured using the Dual-Luciferase Reporter Assay System (Promega, Cat# E1960) and Luminoskan Ascent machine (Thermo Scientific). Renilla luciferase values were normalized to firefly luciferase activity to obtain the relative luciferase activity for plotting.

### Cell and tissue lysis and IB

Cell lysates were prepared using TNT buffer [20 mM Tris (pH 7.5), 150 mM NaCl, 1% Triton X-100] (Beyotime Biotechnology, Cat# P0013) containing a complete protease inhibitor cocktail (Roche, Cat# 4693124001). Mice colon and tumor samples were mechanically homogenized using a homogenizer (Tissuelyser-24, Shanghai Jingxin Experimental Technology) in RIPA buffer [50 mM Tris (pH 7.4), 150 mM NaCl, 1% NP-40, 0.5% deoxycholate, 0.1% sodium dodecyl sulfate (SDS)] containing a complete protease inhibitor cocktail. Cell and tissue lysates were centrifuged at 18,000 × *g* for 15 min at 4 °C and protein concentrations of supernatants were determined by Pierce BCA Protein Assay Kit (Thermo Fisher, Cat# 23225) according to the manufacturer’s instructions. Samples were boiled in 4× Loading buffer [120 mM Tris (pH 6.8), 4% SDS, 20% glycerol, 200 mM dithiothreitol, and 0.05% bromophenol blue] for 5 min at 100 °C. Proteins were separated by 8–15% SDS-polyacrylamide gel electrophoresis and transferred to nitrocellulose membrane (Pall Corporation, Cat# 66485). The membranes were blocked in 5% milk in TBST [10 mM Tris-HCl (pH 8.0), 150 mM NaCl, and 0.05% Tween-20] at room temperature, and then probed with specific primary and HRP-conjugated secondary antibodies. Protein bands were detected using ECL Western Blotting Substrate (Advansta, Cat# K-12045-D50), and the intensity of protein bands was quantified using the Image J software.

### Co-immunoprecipitation

Cells or fresh tissues were suspended and homogenized in TNT buffer containing complete protease inhibitor cocktail, followed by rotation for 15 min at 4 °C. The lysates were centrifuged at 18,000 × *g* for 15 min at 4 °C. Protein concentrations were determined using Pierce BCA Protein Assay Kit (Thermo Fisher) and adjusted to the final volume of 800 μL at a concentration of 1 mg/mL using TNT buffer. Samples of 30 μL of supernatants mixed with 10 μL of 4× Loading buffer were boiled for 5 min at 100 °C as total input. The other supernatants were incubated with 2 μg of specific antibodies overnight at 4 °C and then with 20 μL of Protein G beads (Millipore, Cat# 16-266) or Protein A/G Magnetic Beads (MCE, Cat# HY-K0202) for 1 h. The beads were washed five times with TNT buffer and boiled in 20 μL of 2× Loading buffer for 5 min at 100 °C. IB was applied to detect protein interactions and expressions.

### Re-co-immunoprecipitation

Cell lysates were incubated with anti-ID1 or anti-Flag antibodies overnight at 4 °C and then with 20 μL of Protein G Agarose Beads (Millipore) for 1 h. The beads were washed five times with TBS buffer [10 mM Tris (pH 7.4), 150 mM NaCl], and then incubated with 100 μL of ID1 blocking peptide solution (250 ng/μL) or 3× Flag peptide (250 ng/μL) on an orbital shaker at 4 °C for 1 h. The supernatant (eluted fraction) was collected and used for a second round of immunoprecipitation using specific antibodies. IB was applied to detect protein interaction and expression.

### Real-time quantitative reverse transcription-PCR

Total RNA was isolated from cells using TRIeasy Kit (Yeasen, Cat# 10606ES60) according to the manufacturer’s instructions. RNA (1 µg) was reverse transcribed into cDNA using Hifair™ II 1st Strand cDNA Synthesis SuperMix Kit (Yesen, Cat# 11120ES60) according to the manufacturer’s instructions. Real-time PCR was performed using Hieff® qPCR SYBR Green Master Mix Kit (Yesen, Cat# 11202ES03) and AriaMx (Agilent) machine. The following PCR conditions were used: 95 °C/5 min (1 cycle); 95 °C/10 s; and 60 °C/30 s (40 cycles). glyceraldehyde 3-phosphate dehydrogenase mRNA was normalized to the examined mRNA levels using the 2^−ΔΔCT^ method to obtain the relative mRNA levels. The specific qPCR primer sequences can be found in Supplementary Table 2.

### Chromatin immunoprecipitation

ChIP assay was performed using Pierce™ Magnetic ChIP Kit (Thermo Fisher, Cat# 26157) according to the manufacturer’s instructions. Briefly, chromatin from formaldehyde-fixed cells was fragmented to a size range of 200–500 bases using a Scientz-II D (Ningbo Scientz Biotechnology). Solubilized chromatin was immunoprecipitated with anti-Smad3 antibody (Abcam, ab208182) overnight at 4 °C in IP buffer provided in the ChIP Kit. Antibody–chromatin complexes were pulled down by protein G-Dynabeads, and then eluted by Elution buffer. After crosslink reversal and digestion by RNase A and proteinase K, immunoprecipitated DNA was collected using DNA Clean-Up Column. DNA was quantified by quantitative PCR using fast SYBR Green Master Mix (Yeasen, Cat# 11202ES03). PCR primers used were provided in Supplementary Table 2.

### Protein turnover rate assay

The turnover of ID1 protein was evaluated by CHX chase assay. Cells were seeded into a 12-well plate (5 × 10^5^ cells/well) and treated with CHX (10 μM) (MCE, Cat# HY-12320) in serum-free medium for the time indicated. Cells were washed with phosphate-buffered saline PBS and lysed in TNT buffer containing a complete protease inhibitor cocktail for 15 min on ice. Protein expressions were analyzed by IB. The intensity of immunoblot bands of ID1 and β-actin were quantified using the Image J Software (Media Cybernetics, http://forums.mediacy.com/categories/image-pro-plus-download-install, v1.52) and a ratio of ID1 to its β-actin band intensity was calculated. The final ID1 protein turnover rate at each time point was the percentage of ID1/β-actin at *t* = 0 of each experimental group.

### Protein in vivo poly-ubiquitylation assay

Cells were treated with MG132 (20 μM) (Sigma-Aldrich, Cat# C2211) for 2 h and then suspended in RIPA buffer containing *N*-ethylmaleimide (10 mM) (Sigma-Aldrich, Cat# E3876) and a complete protease inhibitor cocktail (Roche). Soluble lysates were denatured by 1% SDS at 95 °C for 5 min and then diluted ten times by RIPA buffer. Ubiquitylated proteins were immunoprecipitated using antibodies for specific proteins for 4 h and then 20 μL of Protein G beads (Millipore) for 1 h at 4 °C. The beads were washed five times with RIPA buffer and boiled in 20 μL of 2× Loading buffer. IB was applied to detect protein ubiquitylation using anti-ubiquitin antibody.

### Smad3 in vivo mono-ubiquitination assay

LS174T cells were suspended in TNT buffer containing *N*-ethylmaleimide (10 mM) and complete protease inhibitor cocktail followed by rotation for 15 min at 4 °C. Cell lysates were centrifuged at 18,000 × *g* for 15 min at 4 °C. Ubiquitylated proteins were immunoprecipitated with specific antibodies for 4 h and then 20 μL of Protein G beads (Millipore) for 1 h at 4 °C. The beads were washed five times with TNT buffer and boiled in 20 μL of 2× Loading buffer. IB was applied to detect protein ubiquitylation.

### CRISPR genome editing

Nur77- and ID1-knockout cell lines were generated using CRISPR-Cas9 methods^65^. Briefly, the design of single guide RNAs (sgRNAs) was based on recommendations from the Zhang laboratory website (http://crispr.mit.edu/). sgRNAs were prepared for annealing and cloning of sgRNAs into the hCas9 expression plasmid (pX330-U6-Chimeric) as described^66^. The DNA oligonucleotides for sgRNA can be found in Supplementary Table 2.

### Generation and maintenance of cell lines

SW620/sh-control and SW620/sh-Nur77 stable cells were generated using puromycin selection. pSuper-H1 or pSuper-Nur77 was transfected into SW620 cells using Lipofectamine 2000 transfection reagent. Transfected SW620 cells were then treated with puromycin (1 μg/mL) for 1 week and the efficiency of Nur77 knockdown was examined by IB. Puromycin was used at 1 μg/mL to maintain selection pressure on stably transfected SW620 cells. The insertion DNA sequences in pSuper-Nur77 can be found in Supplementary Table 2.

LS174T/control, LS174T/*NR4A1*^−/−^, LS174T/*ID1*^−/−^, and LS174T/*ID1*^−/−^ *NR4A1*^−/−^ stable cells were generated using CRISPR/Cas9 technology. Briefly, LS174T cells at 30% confluence were co-transfected with 5 μg of the appropriate sgRNA-containing pX330-U6-Chimeric plasmids and 2 μg of p3 × FLAG-CMV-10 vector using Lipofectamine 2000 transfection reagent for 48 h. Cells were placed under G418 (1 mg/mL) (Sigma-Aldrich, Cat# A1720) selection for 1 week and cell colonies were picked. *ID1* and/or *NR4A1* knockout were confirmed by IB. G418 was used at 1 mg/mL to maintain selection pressure on stably transfected LS174T cells.

### Cell sphere formation assay

Single-cell suspension was prepared in a 6-well ultra-low attachment plate (1 × 10^3^ cells/well) (Corning, Cat# 3471). Cells were cultured in serum-free DMEM medium supplemented with 1× B-27™ Supplement (Gibco, Cat# 17504044), 20 ng/mL epidermal growth factor (SinoBiological, Cat# 10605), 20 ng/mL basic fibroblast growth factor (Novoprotein, Cat# C046), 5 μg/mL insulin (Yeasen, Cat# 40112ES25), 1 μg/mL hydrocortisone (Yeasen, Cat# 40109ES08), and 100 U/mL penicillin–streptomycin. After 7 days of culture, the number of cell spheres was counted under a microscope (Zeiss) and sphere diameters were measured by the AxioVision software (Zeiss)^67^.

### Histology and immunohistochemistry

Tumors with adjacent tissues of the liver and spleen were processed for paraffin section using standard protocols as previously described^28^. Briefly, tissues were fixed with 4% paraformaldehyde overnight at 4 °C and dehydrated with graded ethanol and xylene. The dehydrated tissues were embedded by paraffin on a tissue embedding machine (Leica) and then cut into 5-μm-thick slices by a microtome slicer (Leica). The paraffin-embedded slices were deparaffinized through xylene and graded ethanol followed by soaking in water to wash the residual ethanol.

For hematoxylin and eosin (H&E) staining, deparaffinized sections of tissues were stained with hematoxylin (Zsbio, Cat# ZLI-9610) for 2 min followed by running water for 6 min. Sections were exposed to acid alcohol (1% HCl) differentiation solution for 30 s followed by running water for 6 min. Sections were then stained with eosin (Zsbio, Cat# ZLI-9613) for 2 min followed by running water for 6 min. Sections were dehydrated by graded ethanol and xylene and fixed by neutral balsam (Sinopharm Chemical Reagent Co., Ltd).

For immunostaining, paraffin-embedded sections were deparaffinized by xylene and/or ethanol using standard protocols followed by boiling in sodium citrate buffer six times (1.8 mM citric acid and 8.2 mM sodium citrate in PBS) for 5 min. Sections were blocked by 3% H_2_O_2_ in methanol for 30 min in the dark, glycine (0.1 M in PBS) for 1 h, and then 5% normal goat serum (Zsbio, Cat# ZLI-9056) for 1 h at room temperature. Sections were then incubated with primary antibodies (1:100) diluted in 1% bovine serum albumin overnight at 4 °C, followed by incubation with secondary antibodies (Zsbio, Cat# PV-9001 and Cat# PV-9002) according to the manufacturer’s instructions. After each incubation, sections were soaked in PBS five times for 3 min. Sections were exposed to DAB (Zsbio, Cat# ZLI-9017) for 20 s–5 min followed by dehydration with xylene and/or ethanol and fixed with neutral balsam.

### Colon cancer tissue array and analysis

All paraffin-embedded arrays containing colon cancer samples (*n* = 80) were serial sections and used for routine immunohistochemical staining (p-Smad3 (S423/425), Nur77, ID1, and Smad4). Immunohistochemical staining of the colon cancer sections was analyzed by the Image-Pro Plus 6.0 photogram analysis system (IPP 6.0, Media Cybernetics, Bethesda, MD, USA)^68^. Briefly, the integrated option density (IOD) of sections with p-Smad3-, Nur77-, or ID1-positive staining and the area of blank staining was counted by Image-Pro Plus 6.0 photogram analysis system. The value of IOD/(total staining area−blank staining area) was considered as the final score of immunostaining intensity.

### Colon cancer liver metastasis model

SW620/sh-ctr and SW620/sh-Nur77 cells at 80% confluence were harvested and resuspended in saline at a density of 2 × 10^6^ cells/mL^69^. Athymic immunodeficient nude mouse was anesthetized with isoflurane by inhalation and then placed on a sterilized and warm operating table. The spleen was exteriorized through a left flank incision. Cells (1 × 10^5^) were slowly injected into the splenic pulp through a 26-gauge needle over 1 min followed by stanched with a sterilized cotton for 3 min (Day 1). All mice were sacrificed when the first mouse appeared lethargic and an enlarged liver could be palpated (Day 28). The livers and spleens were excised and weighed. Spleen coefficient (spleen weight/body weight) and liver coefficient (liver weight/body weight) were analyzed to reflect tumor growth rates^70^. Fresh tumor sections were homogenized in TNT buffer containing a complete protease inhibitor cocktail for determining protein expressions and interactions by IB and co-immunoprecipitation, respectively. Tumors with adjacent spleen or liver tissues were fixed by 4% paraformaldehyde and used for H&E staining.

### Mouse xenograft assay

Briefly, LS174T and LS174T/*ID1*^−/−^ cells (1 × 10^6^) suspended in saline were injected subcutaneously into left and right flanks of the same nude mice, respectively. Similarly, LS174T/*NR4A1*^−/−^ and LS174T/*ID1*^−/−^*NR4A1*^−/−^ cells were injected into another group mouse. After 10 days of inoculation, mice of each xenotransplantation were randomly divided into two groups (*n* = 5) (Day 1). Intraperitoneal injection of oxaliplatin (5 mg/kg, diluted in water) daily was performed. Mice weight was measured by electronic balance daily. Tumor size was measured with Vernier caliper, and the maximum longitudinal diameter (a) and the maximum transverse diameter (b) were determined. The tumor volume was calculated according to the caliper measurement with the ellipsoid formula [tumor volume (mm^3^) = *a* × *b*^2^/2]. Mice were sacrificed after 12 days of treatment and the xenograft tumors were dissected and weighed. Fresh tumor sections were homogenized in TNT buffer containing a complete protease inhibitor cocktail for determining protein expressions by IB.

### Statistical analysis and reproducibility

All data were presented as the mean ± SD of three technical replicates. Two-tailed unpaired Student’s *t* test, one-way analysis of variance (ANOVA) and two-way ANOVA followed by Tukey’s multiple comparisons test were used for statistical analysis using the GraphPad Prism 8.0 software. For all statistical analysis, *p* values <0.05 were considered statistically significant. Each experiment was independently repeated at least two to three times with similar results.

### Reporting summary

A reporting summary for this article is available.

## Supplementary information

Supplementary Information

Reporting Summary

## Data Availability

Data supporting the findings of this study are available within the article and its Supplementary information files, and from the corresponding author upon reasonable request. Source data are provided with this paper.

## Source data

Source Data
