# Supplementary material for: Interplay between transforming growth factor-β and Nur77 in dual regulations of inhibitor of differentiation 1 for colonic tumorigenesis
Source: Nat Commun. 2021 May 14;12:2809. doi: 10.1038/s41467-021-23048-5 (PMC8121807; doi:10.1038/s41467-021-23048-5)
Supplement: Supplementary file 1 — Supplementary Information [file 41467_2021_23048_MOESM1_ESM.pdf]

Supplementary Information

Interplay between transforming growth factor-β and Nur77 in dual regulations of inhibitor of differentiation 1 for colonic tumorigenesis

Boning Niu[1], Jie Liu[1], Ben Lv[1], Jiacheng Lin[2], Xin Li[1], Chunxiao Wu[1], Xiaohua Jiang[2], Zhiping Zeng[1], Xiao-kun Zhang[1], and Hu Zhou[1]*

[1]School of Pharmaceutical Sciences, Fujian Provincial Key Laboratory of Innovative Drug Target Research, High Throughput Drug Screening Platform, Xiamen University, Xiamen, Fujian 361102, China
[2]School of Biomedical Sciences, The Chinese University of Hong Kong, Hong Kong 999077, China
*Correspondence: huzhou@xmu.edu.cn

Supplementary Figures 1 – 8
Supplementary Tables 1 – 2

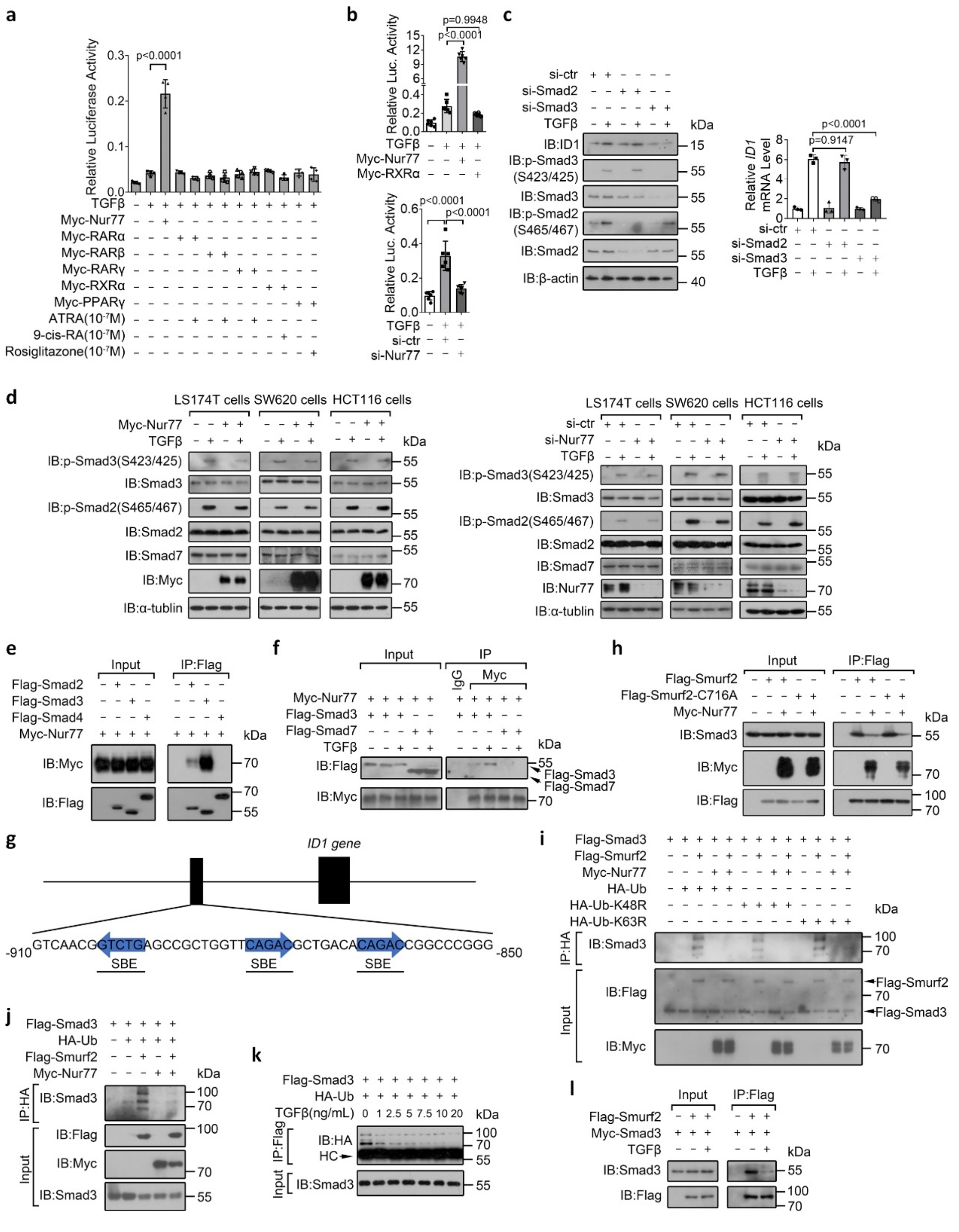

Supplementary Figure 1. Nur77 transcriptionally upregulates TGFβ-induced ID1 expression through inhibiting Smurf2-mediated mono-ubiquitylation of Smad3.
a, b HEK293T (**a**) and HCT116 (**b**) cells transfected with CAGA-luciferase reporter and renilla plasmids together with the indicated expression plasmids or siRNAs were treated with TGFβ (10 ng/mL) and the indicated compounds for 12 h. Cells were harvested, and firefly and renilla luciferase activities were measured. Renilla luciferase values were normalized to firefly luciferase activity and plotted as relative luciferase activity. Luc.: Luciferase; ATRA: All-trans retinoic acid; 9-cis-RA: 9-cis retinoic acid; si-ctr: control siRNA; si-Nur77: Nur77 siRNA. One-way ANOVA followed by Tukey's

multiple comparisons test was used for statistical analysis, and data are presented as means ± SD (**a**, n=4; **b**, n=6; biologically independent samples).

c LS174T cells were transfected with the indicated siRNAs for 48 h followed by TGFβ (10 ng/mL) treatment for 1 h. Protein and ID1 mRNA expressions were examined by immunoblotting (IB) and qRT-PCR, respectively. si-Smad2: Smad2 siRNA; si-Smad3: Smad3 siRNA. Two-way ANOVA followed by Tukey's multiple comparisons test was used for statistical analysis, and data are presented as means ± SD (n=3 biologically independent samples).

d SW620, HCT116 and LS174T cells were transfected with the indicated expression plasmids or siRNAs followed by TGFβ (10 ng/mL) treatment for 1 h. Protein expressions were examined by immunoblotting.

e, f HCT116 cells were transfected with the indicated expression plasmids followed by TGFβ (10 ng/mL) treatment for 1 h. Protein interactions were examined by co-immunoprecipitation (co-IP).

g Nucleotide sequence of TGFβ-responsive region in *ID1* promoter. Smad binding elements (SBE) are indicated by blue arrows.

h, l LS174T cells transfected with the indicted expression plasmids were treated with TGFβ (10 ng/mL) for 1 h followed by co-immunoprecipitation assay of protein interactions.

i-k LS174T cells transfected with the indicated expression plasmids were treated with TGFβ at the indicated doses for 1 h. Smad3 ubiquitylation was examined by immunoprecipitation and immunoblotting using specific antibodies. Ub: Ubiquitin; HC: Heavy chain.

Data represent at least two independent experiments. Source data are provided as Source Data file.

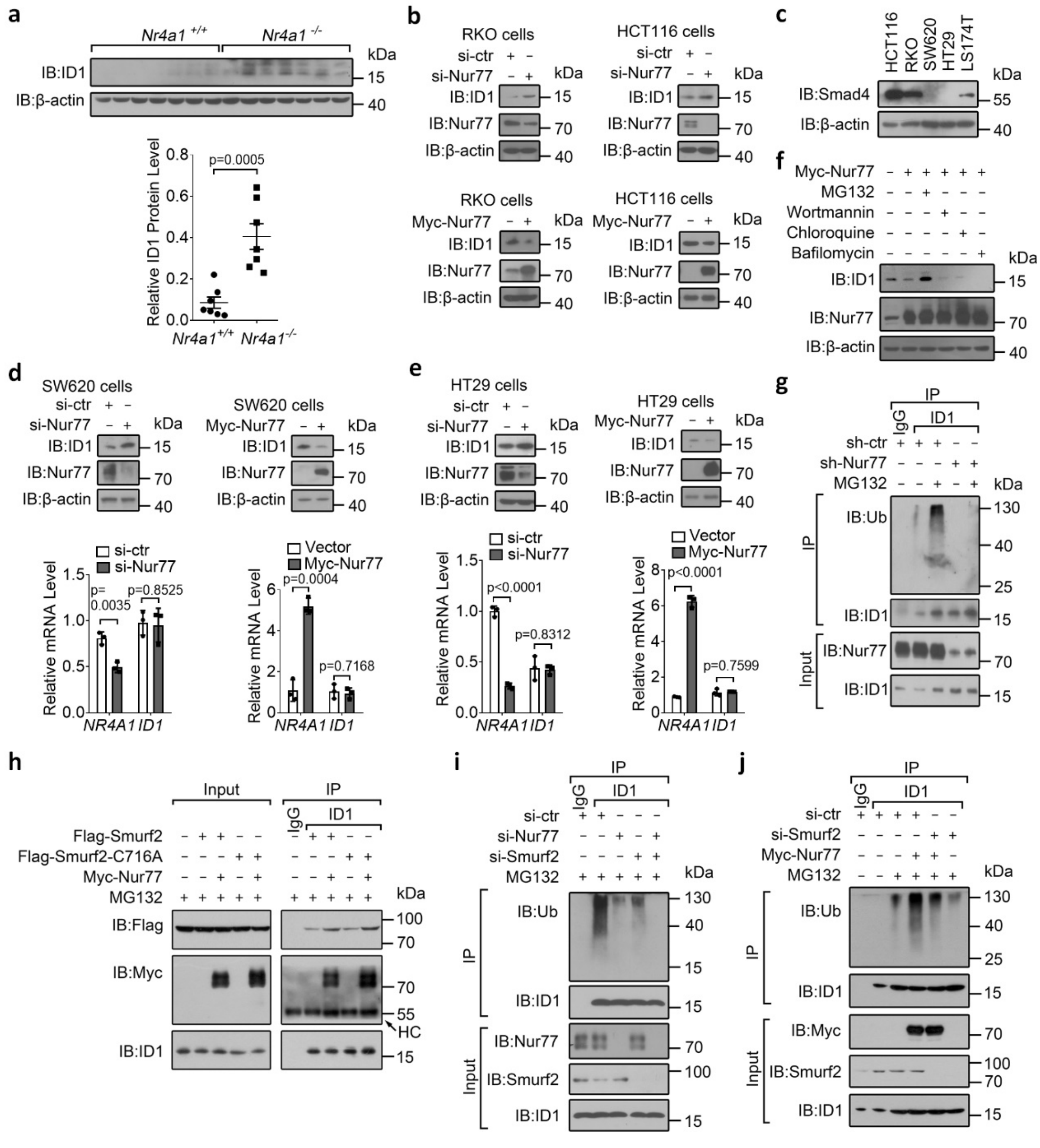

Supplementary Figure 2. Nur77 post-translationally down-regulates ID1 through mediating its association with and ubiquitylation by Smurf2.

a ID1 protein levels in normal colon tissues from *Nr4a1*$^{+/+}$ and *Nr4a1*$^{-/-}$ mice were examined by immunoblotting (IB) and quantified. Two-tailed unpaired Student's *t* test were used for statistical analysis, and data are presented as means ± SD (n=7 mice per group).

b Cells were transfected with Myc-Nur77 expression plasmid or Nur77 siRNA. Protein expression were analyzed by immunoblotting. si-ctr: control siRNA; si-Nur77: Nur77 siRNA.

c Smad4 expression was examined in colon cancer cell lines by immunoblotting.

d, e SW620 (**d**) and HT29 (**e**) cells were transfected with Myc-Nur77 expression plasmid or Nur77 siRNA. Protein and mRNA expressions were examined by immunoblotting and qRT-PCR, respectively. Two-tailed unpaired Student's *t* test were

used for statistical analysis, and data are presented as means ± SD (n=3 biologically independent samples).

f SW620 cells transfected with Myc-Nur77 expression plasmid were treated with MG132 (20 μM), wortmannin (100 nM), chloroquine (10 μM) or bafilomycin (10 nM) for 2 h followed by immunoblotting analysis of protein expression.

g, i, j SW620 cells transfected with the indicated shRNAs, siRNAs or expression plasmids were treated with MG132 (20 μM) for 2 h. ID1 ubiquitylation was examined by immunoprecipitation (IP) using anti-ID1 antibody and immunoblotting using anti-ubiquitin (Ub) antibody. sh-ctr: control shRNA; sh-Nur77: Nur77 shRNA; si-Smurf2: Smurf2 siRNA.

h LS174T cells transfected with the indicated expression plasmids were treated with MG132 (20 μM) for 2 h. Protein interactions were examined by co-immunoprecipitation. HC: Heavy chain.

Data represent at least two independent experiments. Source data are provided as Source Data file.

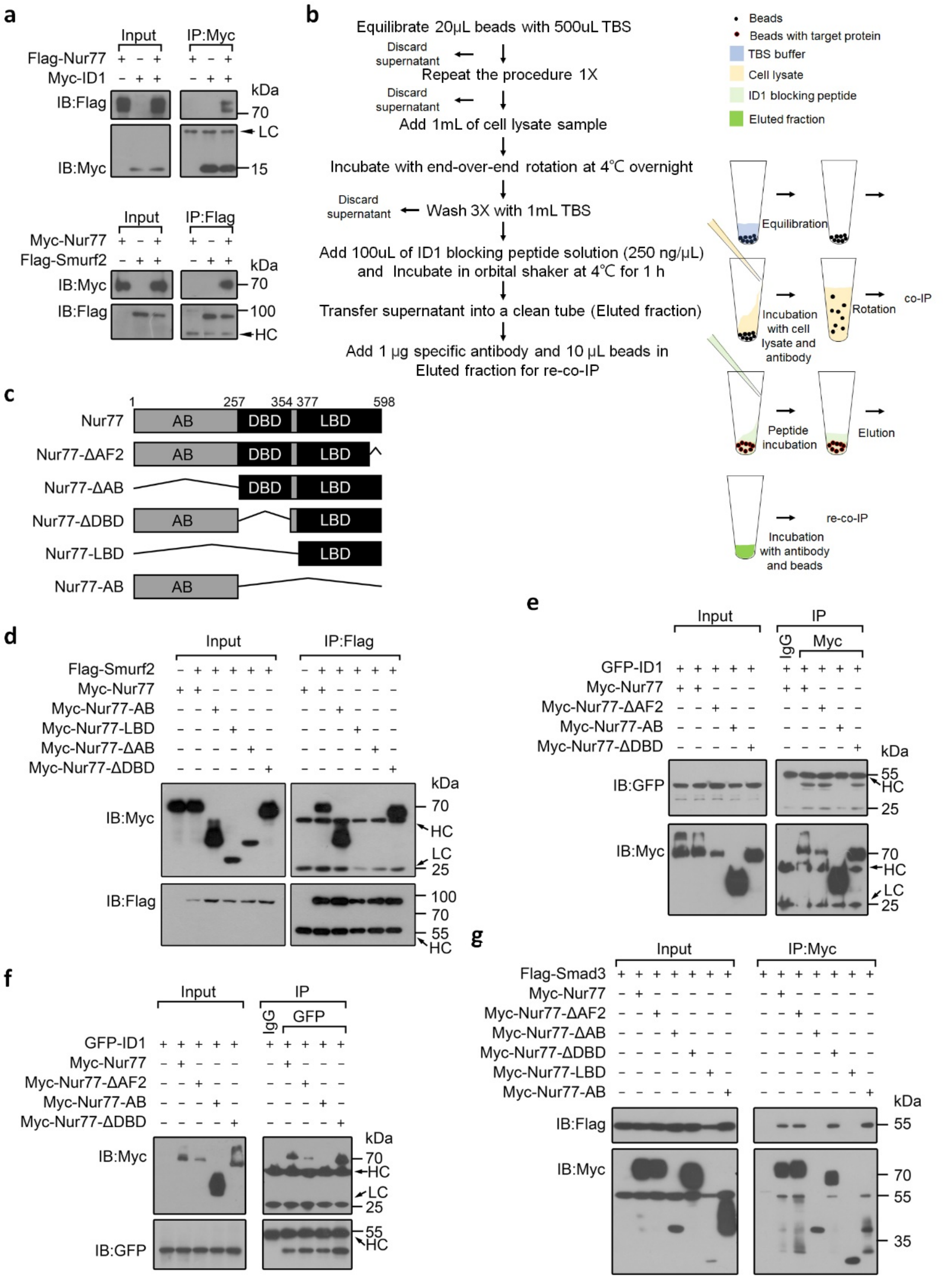

Supplementary Figure 3. Molecular mechanisms underlying Nur77's effects on the interactions of Smurf2 with Smad3 and ID1.

a SW620 cells were transfected with the indicated expression plasmids. Co-immunoprecipitations (co-IP) were performed to examine protein interactions. LC: Light chain; HC: Heavy chain.

b Experimental procedure of re-co-immunoprecipitation assay. TBS indicated TBS buffer [10 mM Tris (pH 7.4), 150 mM NaCl].

c Schematic representation of Nur77 deletion mutants.
d-g HCT116 cells were transfected with the indicated expression plasmids, and the protein interactions were examined by co-immunoprecipitation.
Data represent at least two independent experiments. Source data are provided as Source Data file.

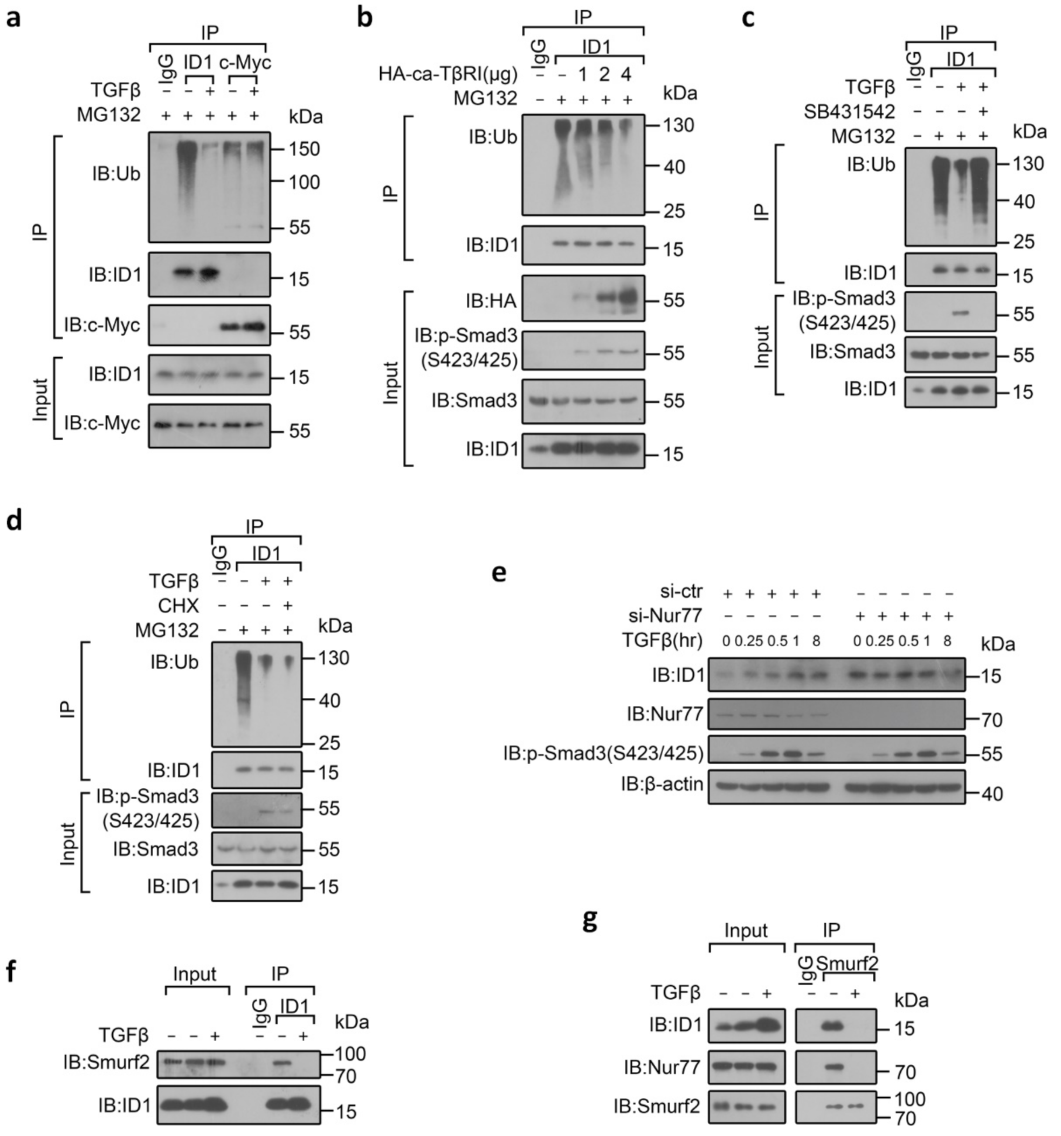

Supplementary Figure 4. TGFβ stabilizes ID1 protein through preventing its Nur77-mediated interaction with and ubiquitylation by Smurf2.

a SW620 cells pretreated with MG132 (20 μM) for 2 h were then treated with TGFβ (10 ng/mL) for 1 h. Protein ubiquitylation was examined by immunoprecipitation (IP) using anti-ID1 or anti-c-Myc antibody followed by immunoblotting using anti-Ubiquitin (Ub) antibody.

b SW620 cells were transfected with indicated amount of HA-ca-TβRI expression plasmids. ID1 ubiquitylation assay was performed. ca, constitutively active.

c SW620 cells were treated with SB431542 (10 μM) for 2 h prior to TGFβ (10 ng/mL) treatment for 1 h. ID1 ubiquitylation assay was performed.

d SW620 cells were treated with cycloheximide (CHX, 10 μM) and/or TGFβ (10 ng/mL) for 1 h. ID1 ubiquitylation assay was performed.

e HT29 cells were transfected with control siRNA or Nur77 siRNA before treatment with TGFβ (10 ng/mL) for the indicated times. Protein expressions were examined by immunoblotting.

f, g SW620 cells were treated with TGFβ (10 ng/mL) for 1 h before co-immunoprecipitation analysis of protein interactions.

Data represent at least two independent experiments. Source data are provided as Source Data file.

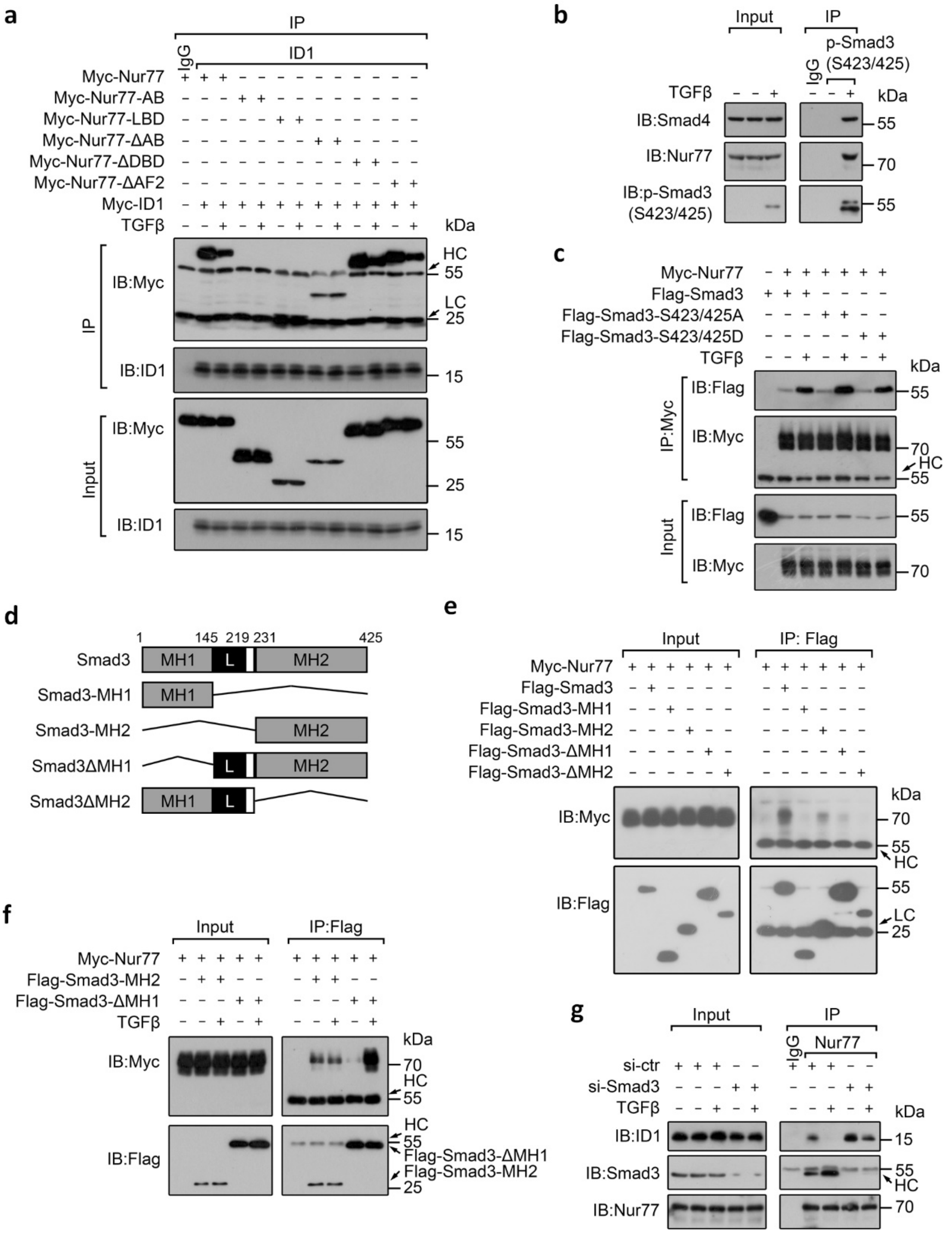

Supplementary Figure 5. TGFβ converts Nur77 role in regulating ID1 expression.

a, b, c, e, f, g HCT116 cells were transfected with the indicated plasmids or siRNAs prior to TGFβ (10 ng/mL) treatment for 1 h. Protein interactions were examined by co-immunoprecipitation (co-IP). LC: Light chain; HC: Heavy chain; si-ctr: control siRNA; si-Smad3: Smad3 siRNA; IB: immunoblotting.

d Schematic representation of Smad3 deletion mutants.

Data represent at least two independent experiments. Source data are provided as Source Data file.

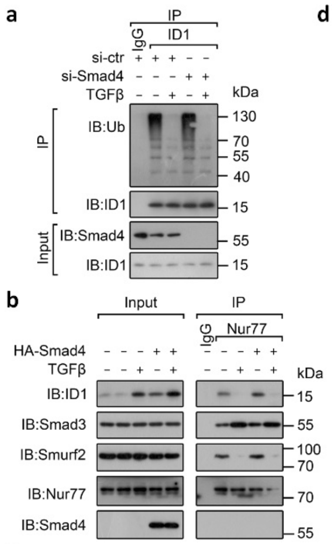

d

No.	Immunostaining Intensity p-Smad3 (S423/425)	ID1	Nur77	No.	Immunostaining Intensity p-Smad3 (S423/425)	ID1	Nur77
1	0.10619140	0.11164020	0.00327389	41	0.03351133	0.62516010	0.57531630
2	0.06257096	0.34118590	0.29863290	42	0.02813615	0.62222150	0.70492470
3	0.01035449	0.22433360	0.57191670	43	0.18723500	0.76724750	0.65274520
4	0.13293710	0.14154790	0.06640781	44	0.07649915	0.68918920	0.37408410
5	0.03415038	0.40227640	0.61441710	45	0.03819469	0.56947920	0.52911580
6	0.00243959	0.32826950	0.55476050	46	0.07453189	0.59301970	0.52525930
7	0.17399250	0.37070830	0.67833890	47	0.03503535	0.57076670	0.49661450
8	0.00062454	0.14895900	0.53077070	48	0.01113828	0.22096460	0.55387180
9	0.04653739	0.16278470	0.44785320	49	0.06034213	0.74655690	0.85211420
10	0.13392460	0.77247180	0.52930250	50	0.03871986	0.59893420	0.56367490
11	0.12173280	0.71761130	0.53965760	51	0.01940405	0.39863600	0.55375350
12	0.00057110	0.35640110	0.38355130	52	0.06022689	0.65077320	0.39657870
13	0.09519143	0.61411890	0.42447650	53	0.00074804	0.08321159	0.64612990
14	0.00324226	0.32659920	0.36912000	54	0.05826531	0.36434560	0.38654340
15	0.00562747	0.26981440	0.54214120	55	0.06085340	0.61865760	0.57009690
16	0.00293874	0.00722171	0.60959560	56	0.00966767	0.01639965	0.75503940
17	0.10532470	0.76643990	0.85426750	57	0.05776349	0.75870130	0.54073770
18	0.02828017	0.74768310	0.81406860	58	0.01231841	0.45114860	0.46947770
19	0.14023130	0.77712460	0.71598800	59	0.03655219	0.65817420	0.55171340
20	0.02260714	0.40244210	0.10197320	60	0.09967080	0.67660320	0.67273870
21	0.01590525	0.53569290	0.34010010	61	0.04200128	0.68415340	0.33766820
22	0.08325326	0.71542930	0.72716300	62	0.00415583	0.42262380	0.42715590
23	0.04337667	0.50898740	0.49935390	63	0.02113668	0.30583820	0.43729480
24	0.01645905	0.04523609	0.61682170	64	0.00101190	0.10080640	0.52623590
25	0.08102365	0.69774310	0.78419910	65	0.04640575	0.41776600	0.44545980
26	0.01597961	0.10934230	0.52311630	66	0.06793031	0.70078860	0.82422120
27	0.06918561	0.69221280	0.54935810	67	0.10032100	0.69420520	0.78809820
28	0.04589998	0.67567070	0.73278420	68	0.01671629	0.03710296	0.61028030
29	0.03590952	0.65402400	0.52772500	69	0.10407560	0.63407360	0.63315840
30	0.05867280	0.60647860	0.57474770	70	0.01886307	0.21950940	0.72465030
31	0.00509033	0.46443410	0.43341700	71	0.00775308	0.30634410	0.68303730
32	0.11693200	0.36084620	0.65176860	72	0.01694975	0.16593580	0.63075440
33	0.05236350	0.60900660	0.57970940	73	0.03104629	0.42258040	0.24901210
34	0.10079690	0.70303310	0.57180080	74	0.06261536	0.52474310	0.63520560
35	0.05839742	0.61107950	0.54120960	75	0.02903273	0.08039578	0.58355130
36	0.04068101	0.60118470	0.64928280	76	0.01462121	0.16658600	0.67540790
37	0.12888370	0.60115640	0.55617540	77	0.00611270	0.06525142	0.54214120
38	0.01322084	0.52658820	0.53463760	78	0.00667424	0.06525142	0.78899230
39	0.08573804	0.56992230	0.64326170	79	0.01266822	0.30295470	0.45371090
40	0.05244985	0.16934230	0.48311630	80	0.00095221	0.35735450	0.67382660

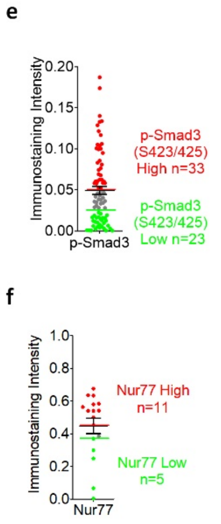

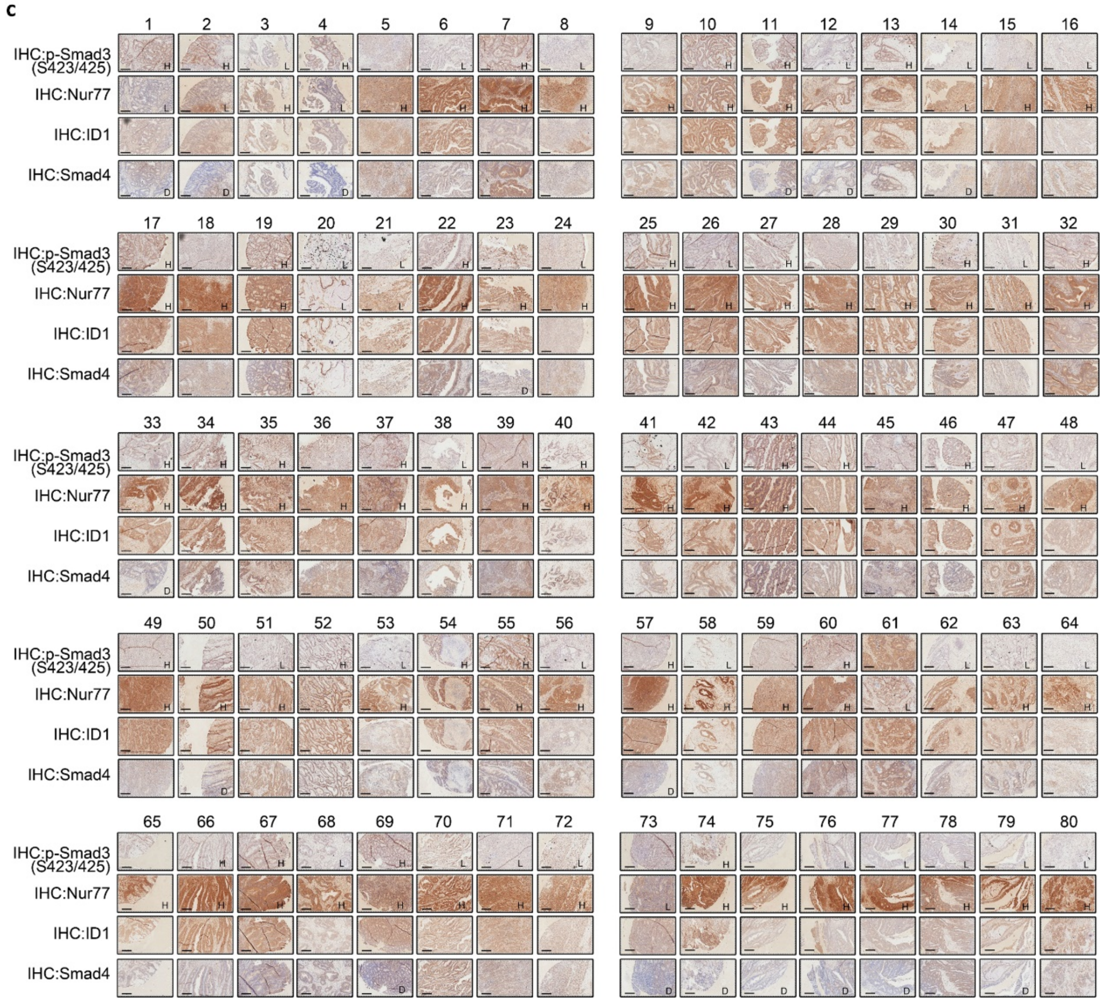

Supplementary Figure 6. Pathophysiological relevance of the TGFβ/Nur77/ID1 axis in colon cancer.

a HCT116 cells were transfected with the indicated siRNAs prior to TGFβ (10 ng/mL) treatment for 1 h. ID1 ubiquitylation assay was performed. si-ctr: control siRNA; si-Smad4: Smad4 siRNA; Ub: Ubiquitin; IP: immunoprecipitation; IB: immunoblotting.

b SW620 cells were transfected with HA-Smad4 expression plasmid prior to TGFβ (10 ng/mL) treatment for 1 h. Protein interactions were examined by co-immunoprecipitation.

c-f ID1, Nur77, p-Smad3 and Smad4 expressions were examined in clinical colon cancer tissues by immunohistochemistry (IHC) analysis using specific antibodies (**c**). Protein immunostaining intensity was quantified by Image-Pro system (**d**). The immunostaining intensity values of p-Smad3(S423/425) higher than 0.5236 and lower than 0.2814 were considered as high and low expression, respectively (**e**). The immunostaining intensity values of Nur77 higher than 0.4334 was considered as high expression (**f**). H: High expression, L: Low expression, D: Deficiency. Scale bars, 200 μm.

Data represent at least two independent experiments. Source data are provided as Source Data file.

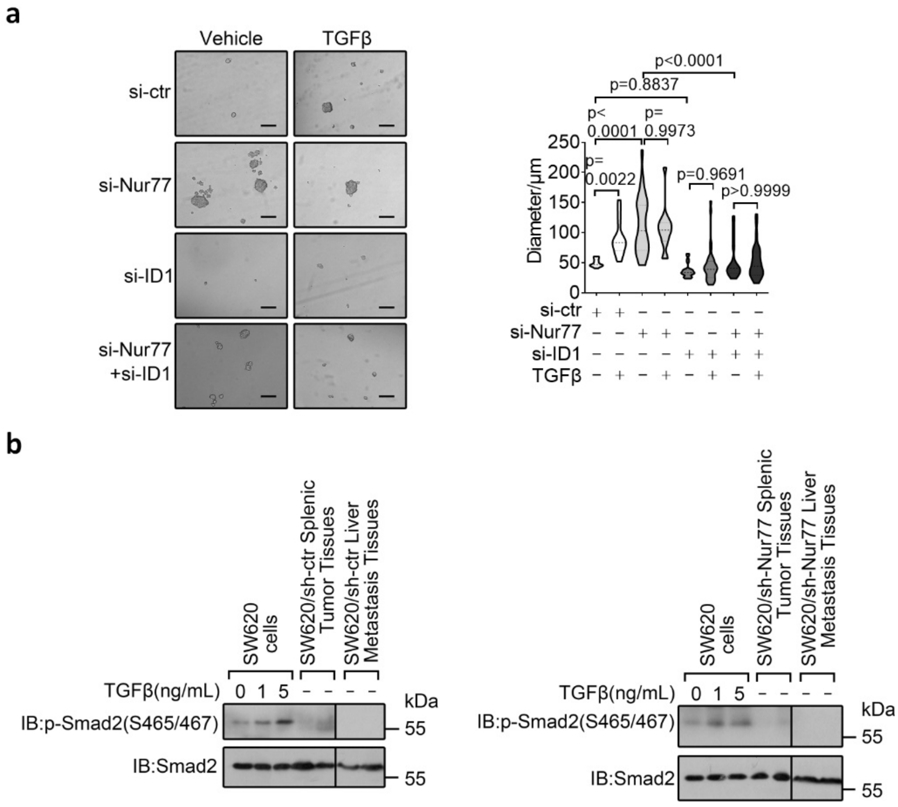

Supplementary Figure 7. Involvement of the TGFβ/Nur77/ID1 axis in colon cancer stemness and metastasis.

a SW620 cells transfected with the indicated siRNAs were cultured for 7 days in the presence or absence of TGFβ (10 ng/mL). Cell spheres were observed by microscope. Represent images were shown and sphere diameters were measured. Scale bars, 200 µm. si-ctr: control siRNA; si-Nur77: Nur77 siRNA; si-ID1: ID1 siRNA. Two-way ANOVA followed by Tukey's multiple comparisons test was used for statistical analysis, and data are presented as means ± SD (n=19, 16, 26, 14, 22, 33, 33, 32, respectively).

b SW620/sh-ctr and SW620/sh-Nur77 cells were injected into spleens of nude mice. Mice were reared for 28 days and then sacrificed. Smad2 phosphorylation status in spleen and liver tumor tissues and in TGFβ-stimulated SW620 cells were examined and compared. sh-ctr: control shRNA; sh-Nur77: Nur77 shRNA.

Data represent at least two independent experiments. Source data are provided as Source Data file.

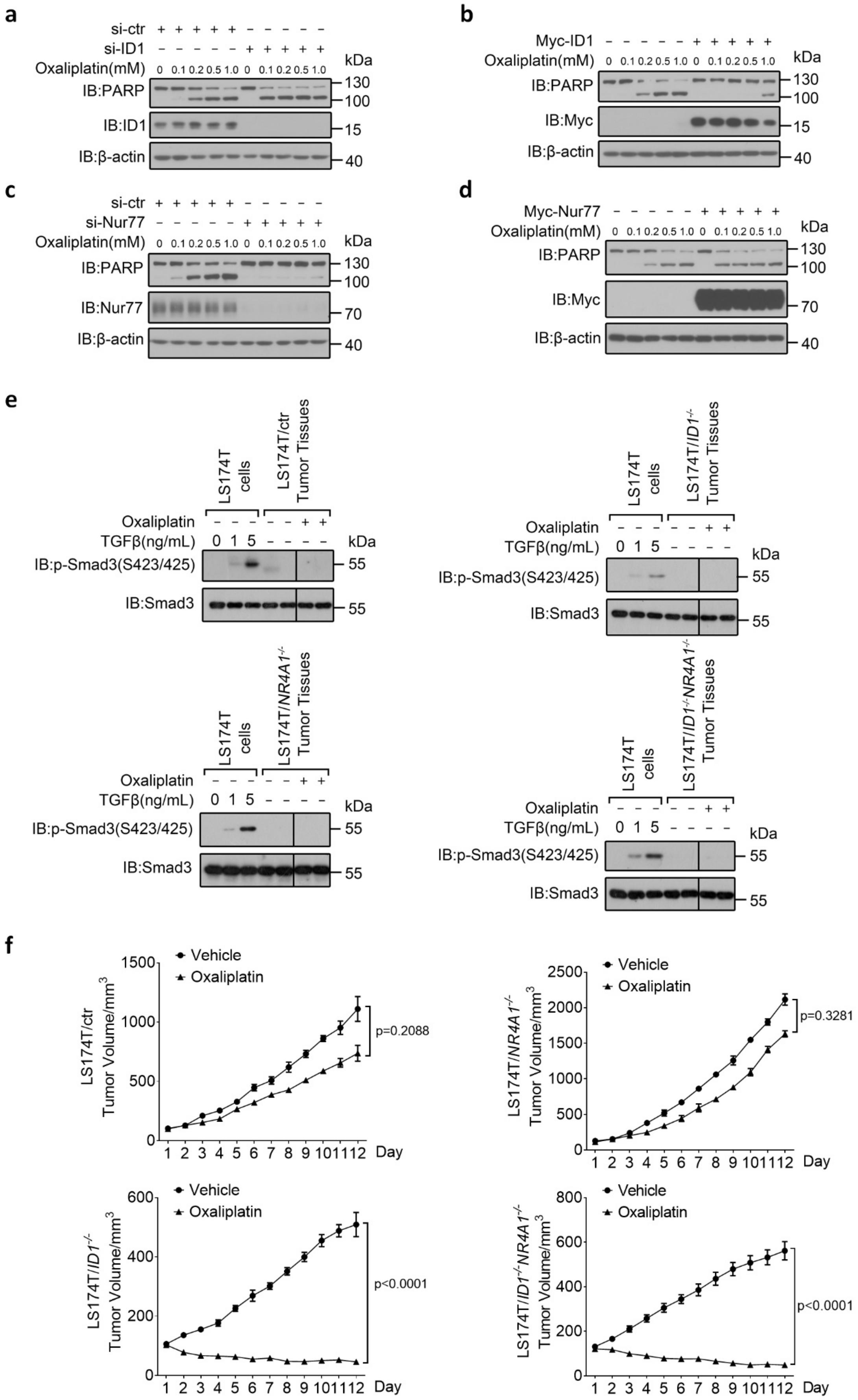

a
si-ctr
si-ID1
Oxaliplatin(mM)
kDa
IB:PARP
IB:ID1
IB:β-actin
b
Myc-ID1
IB:Myc
c
si-Nur77
IB:Nur77
d
Myc-Nur77
e
LS174T cells
LS174T/ctr Tumor Tissues
Oxaliplatin
TGFβ(ng/mL)
IB:p-Smad3(S423/425)
IB:Smad3
LS174T/ID1-/- Tumor Tissues
LS174T/NR4A1-/- Tumor Tissues
LS174T/ID1-/-NR4A1-/- Tumor Tissues
f
Vehicle
Oxaliplatin
LS174T/ctr Tumor Volume/mm3
p=0.2088
Day
LS174T/NR4A1-/- Tumor Volume/mm3
p=0.3281
LS174T/ID1-/- Tumor Volume/mm3
p<0.0001
LS174T/ID1-/-NR4A1-/- Tumor Volume/mm3
p<0.0001

Supplementary Figure 8. Involvement of the TGFβ/Nur77/ID1 axis in colon cancer resistance to oxaliplatin.

a-d LS174T cells were transfected with indicated siRNAs (**a, c**) or expression plasmids (**b, d**). Cells were treated with the indicated doses of oxaliplatin for 12 h. Protein expressions were examined by immunoblotting. si-ctr: control siRNA; si-Nur77: Nur77 siRNA; si-ID1: ID1 siRNA.

e, f The indicated LS174T cell lines were inoculated subcutaneously into flanks of nude mice. After 10 days, mice were intraperitoneally injected with oxaliplatin (5 mg/kg) daily. Visible tumors were measured daily for 12 days (**f**). Mice were then sacrificed. Protein expressions in tumor tissues were examined using the indicated antibodies (**e**). Smad phosphorylating status in tumor tissues was compared to *in vitro* Smad phosphorylation in LS174T cells stimulated by TGFβ (**e**). Two-tailed unpaired Student's *t* test were used for statistical analysis, and data are presented as means ± SD (n=5 mice per group).

Data represent at least two independent experiments. Source data are provided as Source Data file.

Supplementary Table 1. Suppliers for various compounds and agents.

Name	Supplier	Catalog #
Human recombinant TGF-beta1	SinoBiological	10804-HNAC
TRIeasy	Yeasen	10606ES60
SB431542	Sigma- Aldrich	616464
N-Ethylmaleimide	Sigma- Aldrich	E3876
MG132	Sigma- Aldrich	C2211
G418	Sigma- Aldrich	A1720
CHX	MCE	HY-12320
Hexadimethrine bromide	Sigma- Aldrich	H9268
Puromycin Dihydrochloride	MCE	HY-B1743A
B-27™ Supplement (50X), serum free	Gibco	17504044
Basic Fibroblast Growth Factor	Novoprotein	C046
Epidermal Growth Factor	SinoBiological	10605
Insulin	Yeasen	40112ES25
Hydrocortisone	Yeasen	40109ES08
Oxaliplatin	Aladdin	O124003
Wortmannin	Beyotime	S1952
Chloroquine	Sigma- Aldrich	C6628-25G
Bafilomycin	MCE	HY-100558
Nur77 siRNA	Sigma- Aldrich	SASI_Hs02_0 0333289
ID1 siRNA	Sigma- Aldrich	SASI_Hs01_00246328
Smad3 siRNA	Sigma- Aldrich	SASI_Hs01_00208931
Smurf2 siRNA	Sigma- Aldrich	SASI_Hs01_00013161
Control siRNA	Sigma- Aldrich	sc-37007
Pierce™ Magnetic ChIP Kit	Thermo Fisher	26157
Dual-Luciferase ® Reporter Assay System	Promega	E1960
Hifair™ II 1st Strand cDNA Synthesis SuperMix Kit	Yeasen	11120ES60
Hieff® qPCR SYBR Green Master Mix Kit	Yeasen	11202ES03
Protein G Agarose, Fast Flow	Millipore	16-266
Protein A/G Magnetic Beads	MCE	HY-K0202
Pierce BCA Protein Assay Kit	Thermo Scientific	23225
Lipofectamine™ 2000 Transfection Reagent	Thermo Fisher	11668019
Flavopiridol	MCE	HY-10006
ID1 Blocking Peptide	Santa Cruz Biotechnology	SC-133103-P
3xFlag Peptide	Sigma-Aldrich	F4799
Complete™, Mini Protease Inhibitor Cocktail	Roche	4693124001
Nitrocellulose Membrane	Pall Corporation	66485

Supplementary Table 2. Oligonucleotides used for recombinant DNA and qRT-PCR.

Name	Oligonucleotides sequence	Supplier
pSupershNur77 insertion	TCTGGTTCCCTGGACGTTA and<br>TAACGTCCAGGGAACCAGA	Sangon Biotech
Nur77-sgRNA(pX330-Nur77)	5’ -CACCGACCTTCATGGACGGCTACAC - 3’<br>5’ - AAACGTGTAGCCGTCCATGAAGGTC - 3’	Sangon Biotech
ID1-sgRNA(pX330-ID1)	5’ - CACCGAAGGCCGGCAAGACAGCGAG - 3’<br>5’ -AAACCTCGCTGTCTTGCCGGCCTTC - 3’	Sangon Biotech
ID1 ChIP qPCR	5’ - CATGGCGACCGCCCGCGCGG - 3’<br>5’ - GGGCCGGTCTGTGTCAGCGT - 3’	Sangon Biotech
NUR77 qPCR	5’ - ACCCACTTCTCCACACCTTG - 3’<br>5’ - ACTTGGCGTTTTTCTGCACT - 3’	Sangon Biotech
ID1 qPCR	5’-CCGGCAAGACAGCGAGCGGTGCG - 3’<br>5’-GGCGCTGATCTCGCCGTTGAGGG - 3’	Sangon Biotech
GAPDH qPCR	5’ – ACCACAGTCCATGCCATCAC -3’<br>5’ - TCCACCACCCTGTTGCTGTA -3’	Sangon Biotech
BMI1 qPCR	5’ -TGGAGAAGGAATGGTCCACTTC- 3’<br>5’ -GTGAGGAAACTGTGGATGAGGA- 3’	Sangon Biotech
NANOG qPCR	5’ -CAAAGGCAAACAACCCACTT- 3’<br>5’ -TCTGCTGGAGGCTGAGGTAT- 3’	Sangon Biotech
POU5F1 qPCR	5’ -GTCCGAGTGTGGTTCTGTA- 3’<br>5’ -CTCAGTTTGAATGCATGGGA- 3’	Sangon Biotech
SOX-2 qPCR	5’ -ATGGGTTCGGTGGTCAAGT- 3’<br>5’ -GCTCTGGTAGTGCTGGGACA- 3’	Sangon Biotech
LIN28 qPCR	5’ -GCAAAGGTGGTGGAGAAGAG- 3’<br>5’ -GGCTTCCCTCTCGGTTTATC - 3’	Sangon Biotech
