# Supplementary material for: Interplay between transforming growth factor-β and Nur77 in dual regulations of inhibitor of differentiation 1 for colonic tumorigenesis
Source: Nat Commun. 2021 May 14;12:2809. doi: 10.1038/s41467-021-23048-5 (PMC8121807; doi:10.1038/s41467-021-23048-5)
Supplement: Supplementary file 2 — Reporting Summary [file 41467_2021_23048_MOESM2_ESM.pdf]

nature research

Corresponding author(s): Hu Zhou

Last updated by author(s): Apr 22, 2021

Reporting Summary

Nature Research wishes to improve the reproducibility of the work that we publish. This form provides structure for consistency and transparency in reporting. For further information on Nature Research policies, see Authors & Referees and the Editorial Policy Checklist.

Statistics

For all statistical analyses, confirm that the following items are present in the figure legend, table legend, main text, or Methods section.

n/a	Confirmed	
☐	☒	The exact sample size (n) for each experimental group/condition, given as a discrete number and unit of measurement
☐	☒	A statement on whether measurements were taken from distinct samples or whether the same sample was measured repeatedly
☐	☒	The statistical test(s) used AND whether they are one- or two-sided<br>*Only common tests should be described solely by name; describe more complex techniques in the Methods section.*
☒	☐	A description of all covariates tested
☒	☐	A description of any assumptions or corrections, such as tests of normality and adjustment for multiple comparisons
☐	☒	A full description of the statistical parameters including central tendency (e.g. means) or other basic estimates (e.g. regression coefficient) AND variation (e.g. standard deviation) or associated estimates of uncertainty (e.g. confidence intervals)
☐	☒	For null hypothesis testing, the test statistic (e.g. F, t, r) with confidence intervals, effect sizes, degrees of freedom and P value noted<br>*Give P values as exact values whenever suitable.*
☒	☐	For Bayesian analysis, information on the choice of priors and Markov chain Monte Carlo settings
☒	☐	For hierarchical and complex designs, identification of the appropriate level for tests and full reporting of outcomes
☒	☐	Estimates of effect sizes (e.g. Cohen's d, Pearson's r), indicating how they were calculated

Our web collection on statistics for biologists contains articles on many of the points above.

Software and code

Policy information about availability of computer code

Data collection	AriaMx Real-Time PCR System (Agilent Technologies, Peking, China) was used for quantifying mRNA level; Luminoskan Ascent system (Thermo Scientific, Peking ,China) was used for measuring activities of firefly and renilla luciferase; Carl Zeiss Axio Observer A1 system was used for measuring diameter of cell spheres and imaging; Colon cancer tissue arrays were scanned by Superchip company (Shanghai, China); Carl Zeiss Axio Imager AI system was used for imaging H&E staining.
Data analysis	Image J software version 1.52 (NIH) was used for immunoblot intensity acquisition and quantification to obtain ID1 half-life; GraphPad Prism version 8 (GraphPad Software, LaJolla, CA, USA) was used for the preparation of all the graphs and the statistical analyses; Image-Pro Plus version 6.0 (Media Cybernetics, MD, USA) software was used for luminescence measurements of arrays image.

For manuscripts utilizing custom algorithms or software that are central to the research but not yet described in published literature, software must be made available to editors/reviewers. We strongly encourage code deposition in a community repository (e.g. GitHub). See the Nature Research guidelines for submitting code & software for further information.

Data

Policy information about availability of data

All manuscripts must include a data availability statement. This statement should provide the following information, where applicable:

- Accession codes, unique identifiers, or web links for publicly available datasets
- A list of figures that have associated raw data
- A description of any restrictions on data availability

Source data are provided with this paper. The source data underlying all Figs. and Supplementary Figs. are provided as the Source Data file. All the other data supporting the findings of this study are available within the article and its supplementary information files and from the corresponding author upon reasonable request.

Field-specific reporting

Please select the one below that is the best fit for your research. If you are not sure, read the appropriate sections before making your selection.

☒ Life sciences ☐ Behavioural & social sciences ☐ Ecological, evolutionary & environmental sciences

For a reference copy of the document with all sections, see nature.com/documents/nr-reporting-summary-flat.pdf

Life sciences study design

All studies must disclose on these points even when the disclosure is negative.

Sample size	Sample sizes were based on previous experience with similar experimental systems (Hu Zhou et al., Cancer Cell 2010, 17(6). https://doi.org/10.1016/j.ccr.2010.04.023). For in vivo studies, we found 4-6 mice are sufficient to observe significant differences among groups. For detecting ID1 expression and Smurf2/ID1 association in colon tissues derived from Nur77-/- (NR4A1-/-) and wild type mice, we used five animals at least each group. For in vivo colon cancer hepatic metastasis studies, we used four mice for each group. For mouse xenograft assay, we used five mice for each group. For in vitro studies, n=3 or n=4 biologically independent replicates derived from different wells per group were used. This sample size is reported in previous publications to be sufficient for a confident data analysis and in our experience is sufficient to control for technical variations.
Data exclusions	In general, no data were excluded from the analyses unless there were technical problems. For example, mice were dead due to operative injury and were excluded for hepatic metastasis model. Mice were excluded for xenograft studies because of unsuccessful tumor engraftment.
Replication	Every experiments were independently repeated at least two-three times with similar results. Some results were confirmed across multiple cell lines and tumor samples with independent experiments. TGFβ effects were tested at different concentrations in vitro. Some results were confirmed by different investigators. All replication attempts were successful.
Randomization	Nur77-/- (NR4A1-/-) and wild type mice were randomized at same age and collected colon tissues at the same times. For liver metastasis and tumor xenograft studies, mice were randomized in different groups on the basis of body weight before surgical operation. Mice were randomized in different groups on the basis of tumor volumes in a way that each group had nearly equal average tumor volume at the start of treatment. For the other experiments, samples were also randomly allocated to experimental groups.
Blinding	Because we injected different cells into mice to construct tumor metastasis and growth models (Sh-ctr and Sh-Nur77 cells for hepatic metastasis study; control, ID1-/-, NR4A1-/-, and ID1-/-NR4A1-/- cells for xenograft study), the investigators need to label the mice inoculated with different cells correctly. Investigators were not blinded during surgical operation and randomizing the mice in different groups to avoid man-made mistakes. This also ensured animals with nearly equal average tumor volumes, body weight and vitality in each group. However, for in vivo data analysis, investigators were blinded to conditions. All other experiments were not blinded because it was considered unnecessary or not feasible. To avoid man-made operational mistakes, these data were analyzed by individual scientists who carried out the experiments. Although investigators were not blinded to these experiments, data collection was performed in an unbiased manner and the analysis was performed on the endpoints, which is not subject to investigators' bias. Moreover, all data were evaluated independently by at least two investigators.

Reporting for specific materials, systems and methods

We require information from authors about some types of materials, experimental systems and methods used in many studies. Here, indicate whether each material, system or method listed is relevant to your study. If you are not sure if a list item applies to your research, read the appropriate section before selecting a response.

Materials & experimental systems

n/a	Involved in the study
☐	☒ Antibodies
☐	☒ Eukaryotic cell lines
☒	☐ Palaeontology
☐	☒ Animals and other organisms
☒	☐ Human research participants
☒	☐ Clinical data

Methods

n/a	Involved in the study
☒	☐ ChIP-seq
☒	☐ Flow cytometry
☒	☐ MRI-based neuroimaging

Antibodies

Antibodies used	For immunoblotting:<br>Rabbit COL1A1 (1:1000; Santa Cruz Biotechnology SC-8784-R)<br>Rabbit monoclonal Nur77 (1:1000; Cell Signaling Technology 3960)<br>Rabbit monoclonal Smad2 (1:1000; Cell Signaling Technology 5339)<br>Rabbit monoclonal Phospho-Smad2 (Ser465/467) (1:1000; Cell Signaling Technology 3108)

Rabbit monoclonal Smad3 (1:1000; Cell Signaling Technology 9523)
Rabbit monoclonal Phospho-Smad3 (Ser423/425) (1:1000; Cell Signaling Technology 9520)
Rabbit polyclonal Phospho-Smad3 (Thr179) (1:1000; Abcam ab74062)
Rabbit monoclonal p21 Waf1/Cip1 (dilution 1:1000; Cell Signaling Technology 2947)
Rabbit polyclonal c-Myc (1:1000; Santa Cruz Biotechnology SC-789)
Rabbit polyclonal PARP (1:1000; Cell Signaling Technology 9542)
Rabbit polyclonal ID1 (1:1000; Santa Cruz Biotechnology SC-488)
Rabbit polyclonal Ubiquitin (1:1000; Santa Cruz Biotechnology SC-9133)
Mouse monoclonal Ubiquitin (1:1000; Cell Signaling Technology 3936)
Mouse monoclonal ID1 (1:1000; Santa Cruz Biotechnology SC-133104)
Mouse monoclonal Smurf2 (1:1000; Santa Cruz Biotechnology SC-393848)
Mouse monoclonal Smad4 (1:1000; Santa Cruz Biotechnology SC-7966)
Mouse monoclonal Smad7 (1:1000; R&D Systems MAB2029)
Mouse monoclonal β-actin (1:10000; Sigma-Aldrich A5441)
Mouse monoclonal Flag (1:10000; Sigma-Aldrich F1804),
Mouse monoclonal Myc (1:1000; Santa Cruz Biotechnology SC-40),
Mouse monoclonal HA (1:1000; Santa Cruz Biotechnology SC-7392)
Mouse monoclonal Ubiquitin (1:1000; Santa Cruz Biotechnology SC-8017)
Goat Anti-Mouse IgG F(ab')2 Secondary Antibody, HRP conjugate (1:10000; Pierce Chemical 31436)
Goat anti-Rabbit IgG F(ab')2 Secondary Antibody, HRP conjugate (1:10000; Pierce Chemical 31461)
EasyBlot anti Mouse IgG (HRP) (1:1000; GeneTex GTX221667-01)
EasyBlot anti Rabbit IgG (HRP) (1:1000; GeneTex GTX221666-01)
Peroxidase-conjugated AffiniPure Goat Anti-Rabbit IgG (H+L) (1:10000; Jacksonimmuno 111-035-003)
Peroxidase-conjugated AffiniPure Goat Anti-Mouse IgG (H+L) (1:10000; Jacksonimmuno 115-035-003)

For immunoprecipitation:
Rabbit monoclonal Nur77 (1:100; Cell Signaling Technology 3960)
Rabbit monoclonal Smad3 (1:100; Cell Signaling Technology 9523)
Rabbit monoclonal p21 Waf1/Cip1 (1:50; Cell Signaling Technology 2947)
Rabbit polyclonal c-Myc (1:50; Santa Cruz Biotechnology SC-789)
Mouse monoclonal ID1 (1:50; Santa Cruz Biotechnology SC-133104)
Mouse monoclonal Smurf2 (1:50; Santa Cruz Biotechnology SC-393848)
Mouse monoclonal Flag (1:100; Sigma-Aldrich F1804)
Mouse monoclonal Myc (1:50; Santa Cruz Biotechnology SC-40)
Mouse monoclonal HA (1:50; Santa Cruz Biotechnology SC-7392)

For chromatin immunoprecipitation:
Rabbit monoclonal Smad3 ChIP grade (1:100; Abcam ab208182)

For immunohistochemistry staining:
Rabbit monoclonal Nur77 (1:100; Cell Signaling Technology 3960)
Rabbit monoclonal Phospho-Smad3 (Ser423/425) IHC grade (1:100; Abcam ab52903)
Mouse monoclonal ID1 (1:100; Santa Cruz Biotechnology SC-133104)
Mouse monoclonal Smad4 (1:100; Santa Cruz Biotechnology SC-7966)

Validation

All the antibodies used are commercially available. The antibodies for a specific species or application have been confirmed by manufacturers to be used for that species/application and the related information is available on the website and/or in the antibody datasheet.
For immunoblotting:
Rabbit COL1A1: validated by the manufacturer and the reference (Pei-Wen Wang, et al. 2019, Cell) as well as by our group in SW620 colon cancer cells for immunoblotting application.
Rabbit monoclonal Nur77: validated by the manufacturer using COS-7 cells overexpressed human Nur77 and by our group in colon cancer cells for immunoblotting application.
Rabbit monoclonal Smad2: validated by the manufacturer using HeLa or SMAD2 knockout cells and by our group in SW620, LS174T, HCT116 and HT29 cells for immunoblotting application.
Rabbit monoclonal Phospho-Smad2 (Ser465/467): validated by the manufacturer using TGF-beta treated HeLa and NIH3T3 cells and by our group in colon cancer cells for immunoblotting application.
Rabbit monoclonal Smad3: validated by the manufacturer using HeLa cells or HeLa cells with an apparent in-frame truncation mutation in the gene encoding SMAD3 and by our group in colon cancer cells for immunoblotting application.
Rabbit monoclonal Phospho-Smad3(Ser423/425): validated by the manufacturer using HT-1080, C2C12 and KNRK cells treated with TGF-β (10 ng/ml, 30 min) and by our group in colon cancer cells for immunoblotting application.
Rabbit monoclonal Phospho-Smad3 (Thr179): validated by the manufacturer using HeLa cells treated with TNF (20ng/ml, 2mins) and by our group in colon cancer cells for immunoblotting application.
Rabbit monoclonal p21 Waf1/Cip1: validated by the manufacturer using HeLa and p21 Waf1/Cip1 knockout HeLa cells and by our group in SW620 colon cancer cells for immunoblotting application.
Rabbit polyclonal c-Myc: validated by the manufacturer and reference (Cao, X. et al. 2015. Nat Commun) and by our group in LS174T and SW620 colon cancer cells for immunoblotting application.
Rabbit polyclonal PARP: validated by the manufacturer using staurosporine-treated (1 μM) NIH3T3 and Jurkat cells and by our group in LS174T cells for immunoblotting application.
Rabbit polyclonal ID1: validated by the manufacturer and reference (Shin, J. et al. 2010. Nature).
Rabbit polyclonal Ubiquitin: validated by the manufacturer and reference (Jiang, H. et al. 2013. Cell death & disease).
Mouse monoclonal ID1: validated by the manufacturer using Ramos and HeLa cells and by our group in colon cancer cells for immunoblotting application.
Mouse monoclonal Smurf2: validated by the manufacturer, reference (Liang, C., et al. 2018. Nat. Commun.)
Mouse monoclonal Smad4: validated by the manufacturer using NIH3T3, 3T3-L1, C3H/10T1/2, A-10, HeLa and Jurkat cells and by

our group in Smad4 deficient cells for immunoblotting application.
Mouse monoclonal Smad7: validated by the manufacturer using H9 human cutaneous T lymphoma cell line, PT18 mouse mast/basophil cell line, and Rat-2 rat embryonic fibroblast cell line and by our group in colon cancer cells for immunoblotting application.
Mouse monoclonal β-actin: validated by the manufacturer and by our group in several cells for immunoblotting application.
Mouse monoclonal Flag: validated by the manufacturer and by our group in colon cancer cells overexpressed Flag-tagged proteins for immunoblotting application.
Mouse monoclonal Myc: validated by the manufacturer using HeLa, Jurkat and K-562 cell lysate and by our group in colon cancer cells overexpressed Myc-tagged proteins for immunoblotting application.
Mouse monoclonal HA: validated by the manufacturer using HEK 293T cells with HA-tagged fusion proteins showing N-terminal HA-tagged JNK2 and JNK1 and C-terminal HA-tagged Daxx and by our group in colon cancer cells with HA-tagged proteins for immunoblotting application.
Mouse monoclonal Ubiquitin: validated by the manufacturer using 293T cells transfected with human ubiquitin expression plasmids and by our group in colon cancer cells expressed HA-tagged ubiquitin for immunoblotting application.
Goat Anti-Mouse IgG F(ab')2 Secondary Antibody, HRP conjugate: validated by the manufacturer and by our group in years for immunoblotting application.
Goat anti-Rabbit IgG F(ab')2 Secondary Antibody, HRP conjugate: validated by the manufacturer and by our group in years for immunoblotting application.
EasyBlot anti Mouse IgG (HRP): validated by the manufacturer (Bieging-Rolett KT et al. 2020, Mol Cell), and by our group in years for immunoblotting application.
EasyBlot anti Rabbit IgG (HRP): validated by the manufacturer (Gao S et al. 2020, Nat Genet), and by our group in years for immunoblotting application.
Peroxidase-conjugated AffiniPure Goat Anti-Rabbit IgG (H+L): validated by the manufacturer and by our group in years for immunoblotting application.
Peroxidase-conjugated AffiniPure Goat Anti-Mouse IgG (H+L): validated by the manufacturer and by our group in years for immunoblotting application.

For immunoprecipitation:
Rabbit monoclonal Nur77: validated by the manufacturer using COS-7 overexpressed human Nur77 and by our group (Mengjie Hu, et al., Mole Cell, 2017).
Rabbit monoclonal Smad3: validated by the manufacturer and by our group in colon cancer cells for immunoprecipiation application.
Rabbit monoclonal p21 Waf1/Cip1: validated by the manufacturer and by our group in SW620 cells for immunoprecipitation application.
Rabbit polyclonal c-Myc: validated by the manufacturer and by our group in SW620 cells for immunoprecipitation application.
Mouse monoclonal ID1: validated by the manufacturer and by our group using colon cancer ID1 knock down cells for immunoprecipitation application.
Mouse monoclonal Smurf2: validated by the manufacturer and by our group using colon cancer Smurf2 knock down cells for immunoprecipitation application.
Mouse monoclonal Flag: validated by the manufacturer and by our group using it for Flag-tagged protein immunoprecipitation application for years.
Mouse monoclonal Myc: validated by the manufacturer and by our group using it for Myc-tagged protein immunoprecipitation application for years.
Mouse monoclonal HA: validated by the manufacturer and by our group using it for HA-tagged protein immunoprecipitation application for years.

For chromatin immunoprecipitation:
Rabbit monoclonal Smad3 ChIP grade: validated by the manufacturer using HaCaT cells and by our group in LS174T cells for chromatin immunoprecipitation application.

For immunohistochemistry staining:
Rabbit monoclonal Nur77: validated by the manufacturer and reference (Peng-bo Yang et al., 2020, PNAS).
Rabbit monoclonal Phospho-Smad3 (Ser423/425) IHC grade: validated by the manufacturer for immunohistochemistry staining application.
Mouse monoclonal ID1: validated by the manufacturer using formalin fixed, paraffin-embedded human placenta tissue showing cytoplasmic staining of trophoblastic cells for immunohistochemistry staining application.
Mouse monoclonal Smad4: validated by the manufacturer using formalin fixed, paraffin-embedded human small intestine tissue showing cytoplasmic staining of glandular cells for immunohistochemistry staining application.

Eukaryotic cell lines

Policy information about cell lines

Cell line source(s)

All the cell lines were purchased from American Type Culture Collection (ATCC). Human colon cancer cells HCT116 (ATCC, Cat. No. CCL-247); LS174T (ATCC, Cat. No. CL-188); RKO (ATCC, Cat. No. CRL-2577); SW620 (ATCC, Cat. No. CCL-227); HT29 (ATCC, Cat. No. HTB-38); COLO 205 (ATCC, Cat. No. CCL-222); HCT-15 (ATCC, Cat. No. CCL-225) and kidney cells HEK293T (ATCC, Cat. No. CRL-3216).

Authentication

The cell lines have been validated by the suppliers. The growth of the cell lines used were verified with the supplier's data sheets with stable morphology feature and pharmacological responses. SW620/sh-control and SW620/sh-Nur77 stable cells were generated using puromycin selection. LS174T/control, LS174T/NR4A1-/-, LS174T/ID1-/- and LS174T/ID1-/-NR4A1-/- stable cells were generated using CRISPR/Cas9 technology. All stable cells were validated by STR typing (Genetic testing

	biotechnology, Suzhou, China) and the results showed that SW620/sh-control and SW620/sh-Nur77 were SW620 cells, and LS174T/control, LS174T/NR4A1-/-, LS174T/ID1-/- and LS174T/ID1-/-NR4A1-/- were LS174T cells.
Mycoplasma contamination	All cell lines were negative for mycoplasma contamination.
Commonly misidentified lines (See ICLAC register)	None of the cell lines used are listed in ICLAC database.

Animals and other organisms

Policy information about studies involving animals; ARRIVE guidelines recommended for reporting animal research

Laboratory animals	The NR4A1 gene knock out mice (NR4A1-/- C57BL/6J, Stock No: 006187) and control mice (C57BL/6J, Stock No: 000664) were purchased from the Jackson Laboratory (Bar Harbor, Maine, USA). BALB/C nude mice were purchased from the Charles River (Peking, China). All experiments were performed on female cohorts at 2-months of age. All mice were housed in pathogen-free facilities, in a 12-hour light/dark cycle with temperatures of 21-23°C and 50~55% humidity. Mice were housed together when possible in ventilated cages, with chow and water supply ad libitum.
Wild animals	The study did not involve wild animals.
Field-collected samples	The study did not involve samples collected from the field.
Ethics oversight	All animal experiments were performed in accordance with the National Institutes of Health guide for the care and laboratory animals and with the approval of the Animal Care and Use Committee of Xiamen University. All mice were housed in pathogen-free facilities, in a 12-hour light/dark cycle in ventilated cages, with chow and water supply ad libitum. The mice were monitored daily for signs of health and distress.

Note that full information on the approval of the study protocol must also be provided in the manuscript.
